# Environmental and economic evaluation of Egyptian cement production using refuse-derived fuel from municipal solid waste

**DOI:** 10.1038/s41598-026-44273-2

**Published:** 2026-04-02

**Authors:** Ahmed Ali, Mohamed E. Abuarab, Mohamed M. Ibrahim, Mahmoud A. Abdelhamid, Mohamed A. Refai

**Affiliations:** 1 Agricultural Engineering Department, Faculty of Agriculture, Cairo University, Giza, 12613 Egypt; 2 Agricultural Engineering Department, Faculty of Agriculture, Ain Shams University, 11241 Cairo, Egypt

**Keywords:** Cement production, Economic, Environmental impacts, Life cycle assessment, Refused-derived fuel, Municipal Solid Waste, Climate sciences, Engineering, Environmental sciences, Environmental social sciences

## Abstract

Egypt’s cement sector is a major environmental polluter, yet life cycle-based environmental assessments of this industry remain scarce in the country, highlighting a critical research gap that this study addresses. This study employs Life Cycle Assessment (LCA) to evaluate the environmental impacts of cement manufacturing under four Refuse-Derived Fuel (RDF) thermal substitution scenarios, Sc-II (20%), Sc-III (50%), Sc-IV (80%), and Sc-V (100%), in comparison with a conventional baseline scenario (Sc-I). The functional unit is defined as one ton of produced cement, and the system boundary follows a cradle-to-gate approach, encompassing waste collection, RDF processing, fuel substitution, and cement production. The findings demonstrate a consistent and proportional relationship between RDF utilization rate and environmental performance, indicating that higher RDF substitution rates correspond to progressively greater reductions in emissions across the assessed impact categories. Sc-V had the lowest Global Warming Potential (GWP) score of 858.18 kg CO_2_-eq ton^− 1^. As compared to the Sc-I, Sc-V decreased GWP, acidification, eutrophication, photochemical oxidants, and abiotic depletion by 19.1, 38.7, 33.7, 45.2, and 12.9%, respectively. The economic analysis shows that integrating 20% RDF slightly increases capital investment (0.026 to 0.033 $ ton⁻¹) but significantly reduces operating costs by 5.65 $ ton⁻¹, while net revenue remains stable (6.09 $ ton⁻¹). Additionally, RDF substitution saves 0.134 $ ton⁻¹ of cement compared to natural gas and hard coal, confirming that RDF is an economically viable and sustainable alternative fuel for cement production.

## Introduction

The creation of a coherent and efficient waste management approach especially in relation to urban waste is among the key challenges within the context of sustainable development. Societal wealth, buying habits, and economic growth are some of the factors that contribute greatly towards the creation of these wastes. Use of sustainable development principles in waste management requires that a hierarchy of the waste management methodologies be observed. The chain of command covers waste elimination, waste reuse preparation/recycling, participation in different recovery activities, and when necessary, disposal of the waste which cannot be recovered^1^. All these principles have been fundamentalized into the organization of strategies at the European Union as well as the Polish levels with the case examples being the waste framework directive, the landfill directive, and the national waste management plan^1^.

According to the estimates, Egypt produces about 28 million tons of municipal solid waste (MSW) every year, and greater Cairo region represents approximately 40% of total features. In Egypt, the official MSW composition is 56% of organics, 13% plastics, 10% paper and cardboard, 4% glass, 2% metals, and 15% other substances^2^. Depending on the governorate the organic fraction can fall between 41 and 70%. According to the latest statistics, 81% of MSW are discarded through open dumping, 12% of MSW is recycled, but only 7% of MSW is landfilled^2,3^. Considering the indicative expansion of the collected coverage and efficiency of MSW, a considerable part of the collected MSW could be redirected towards the production of RDF, particularly, energy-rich (plastics, paper, and cardboard) portions of the collection^2,4^.

The stages of processing of the MSW into refuse-derived fuel (RDF) include sorting, shredding, magnetic separation, and pelletizing. RDF has significant potential in the energy intensive sectors like cement production whereby it can be used as an alternative energy source albeit as partial substitute of the traditional fossil fuels, coal and heavy fuel oil. This source can not only reduce the dependency on the exhaustible fossil wealth but also help to decrease the emission of gaseous emissions^5,6^.

With the growing population, accompanied by urbanization as well as industrialization, the collection of waste has emerged to be a major issue to the governing bodies as well as to the ecological systems. Thus, there is an essential need to develop sustainable approaches to waste management, especially those where waste would be transformed into a useful resource instead of being placed in a landfill or burnt^7^. Waste to Energy (WtE) represents a more profitable alternative to landfilling of MSW. The sector of conversation on the production of RDF has gained an increasing interest in MSW-based production. This method is usually viewed as a cost effective WtE technology, which is realized by means of producing waste pellets out of inorganic products. Based on the RDF production process, size reduction and magnetic separation are carried out to formulate fluff. Fluff RDF (fRDF) is an additional fuel, it is produced as a result of metals, glass and other dangerous materials extraction out of waste. Next, fRDF may be turned into a densified RDF (dRDF) through the addition of calcium di-hydroxide^8^.

The fuel used should also comply with the set quality standards^9^ which are vital in determining the suitability of the fuel to be used in burning the cement in cement kilns. The critical parameters include calorific value (more than 14 MJ kg^− 1^), moisture level (less than 15%), chlorine level (less than 0.8%), sulfur level (less than 2.5%), ash level (less than 15%), PCB level (less than 50 mg kg^− 1^) and the level of heavy metals (less than 2,500 mg kg^− 1^)^9^. The morphological variability of municipal waste makes the coercion of these criteria a difficult task to undertake. Particularly, in the case of mixed waste in municipalities, even the isolation of the combustible part is not enough to make it possible to be incinerated in a cement kiln^10^. On the other hand, the use of RDFs has significant environmental benefits as it reduces the need to extract, process and burn fossil fuels (Table 1).

**Table 1:** Key energy consumption and CO₂ emission indicators in the cement industry.

Parameter	Value/Information	Source
CO₂ emission per ton cement	0.83 t CO₂ t^− 1^ (80% clinker factor)	^11^
Calcination emissions	0.45 t CO₂	^11^
Fuel combustion emissions	0.28 t CO₂	^11^
Operational emissions	0.10 t CO₂	^12^
Energy use	3.3 GJ t^− 1^ cement	^13^
Fossil fuel share	85–90% of total energy	
Energy cost share	40% of total costs	^11^
Global CO₂ contribution	≈ 7% (2.9 billion t y^− 1^, 2021)	^14^

RDF is another green fuel that is increasingly becoming an option in cement production as an alternative fuel, which helps in supporting the concept of the green economy by avoiding landfills, consuming less of fossil fuels, and lowering greenhouse gas (GHG) emissions. RDF is made out of different waste sources, such as MSW, plastics, textiles, and industrial by-products, and refined to fit the calorific and quality demands of cement kilns^15,16^. The production and utilization of RDF also enhance the recycling cycle of materials and energy particularly in areas with a small landfill area^17–20^. RDF inorganic fraction can be used in cement, which contributes to even more recycling of the materials^18^.

The cement industry is one of the economic and infrastructure pillars of any nation and has been accepted to be a significant sector of the construction industry. Cement is a process that is highly energy-consuming, which means that close to 3.3 GJ of energy is required to produce a 1 ton of cement^15,21^. Much of this energy demand, about 85% to 90% of the total, is met by the burning of fossil fuels. The cement industry contributes to the amount of carbon dioxide (Globally) consisting of about 7% (∼2.9 billion tons of CO_2_ y^− 1^ in 2021). The role that cement industry has played in climate change and the imminent need to curb mitigation measures have been highlighted^22^. The main three sources of CO_2_ emissions in the cement production process include calcination (50%), fuel burning in the kiln (40%), and manufacturing (10%)^23^.

Under approximately launched Indonesian climate change initiative (called RAN-GRK), cement is one of the priority areas that participate in strategies and implementation of GHG emission cuts, together with the metals industry. According to Chaves, Siman^15,21^, the GHGs caused by the cement manufacturing within the Chinese population are predominantly due to the burning of fossil fuels within the manufacturing process, which contributes to a high percentage of about 86% of all the CO_2_ emissions. A one ton of cement product yields 0.83 tons of CO_2_ at an 80% content clinker product. These emissions include 0.45 ton of CO_2_ during the process of calcification, 0.28 ton of CO_2_ during the process of coal burning, and 0.1 ton of CO_2_ in electrical power generation to run the plant itself^11,12^. According to European Commission, in 2010 the cement industry was indicated as the company with the largest need of fuel. Anasstasia, Lestianingrum^11^ reported that 40% of total cost of running a cement plant was devoted to acquiring energy. Increased CO_2_ emissions of the globe means the increased dependence on fossil fuels. In the meantime, the major environmental problem associated with the production of cement is the emissions produced.

Environmental impact analysis of the process of converting MSW to energy in the form of derivation of dRDF may be discussed within the framework of the LCA^24^. LCA is a sort of methodology that is used in the quantification of the environmental outcomes related to a product throughout the entire product life cycle^24^. LCA simulation is based on the standards of ISO 14,040 and ISO 14,044, and it is generally accepted that it is a significant tool needed to plan a waste management system. LCA helps to evaluate the effects on the environment of different alternatives of solutions and helps identify the areas that should be improved dramatically with the help of impact assessment simulation that involves the risks of acidification, emissions of greenhouse gases, toxicity, and photochemical oxidation, and so on. Waste to energy conversion can also result in emissions which are released to the atmosphere as air pollutants^25,26^.

The production of RDF using solid waste left by municipalities to utilize it as a combustion fuel in powder cement industry furnace as an alternative to traditional fossil fuels is a relatively new concept. Despite a rising interest, there are still urgent requirements in terms of more detailed research to consider its environmental, economic, and technical consequences. The effects of RDF in the environmental parameters are contingent on the nature of the municipal solid waste, recovery rate, the procedure of the RDF manufacture, and the heating value among other factors. As everything in RDF depends on the geographical location, the environmental effects and the economic aspects of the production and usage of RDF also can vary, which is why it is vital to investigate this issue, in particular, how the production waste affects conditions in Egypt.

This study aims to: (i) conduct a comprehensive LCA of cement manufacturing in Egypt under five thermal substitution scenarios ranging from conventional fossil fuel combustion (Sc-I) to full RDF substitution (Sc-V: 100%); (ii) evaluate the techno-economic feasibility of RDF integration by analyzing capital investment, operating costs, and net revenue across substitution scenarios; and (iii) provide evidence-based recommendations to support the transition toward more sustainable and economically viable cement production practices in Egypt.

The principal contributions of this study to the existing body of knowledge are threefold. First, it presents one of the first cradle-to-gate LCA studies specifically focused on the Egyptian cement industry, addressing a critical gap in regional environmental literature. Second, it establishes a quantitative link between increasing RDF substitution rates and progressive reductions in environmental emissions, offering a robust scientific basis for policy-oriented decision-making. Third, it demonstrates the economic competitiveness of RDF as an alternative fuel, showing that higher substitution rates yield meaningful reductions in operating costs without adversely affecting revenue, thereby reinforcing the dual environmental and economic case for RDF adoption in Egypt’s cement sector.

## Materials and methods

### Study area

**Fig. 1:**
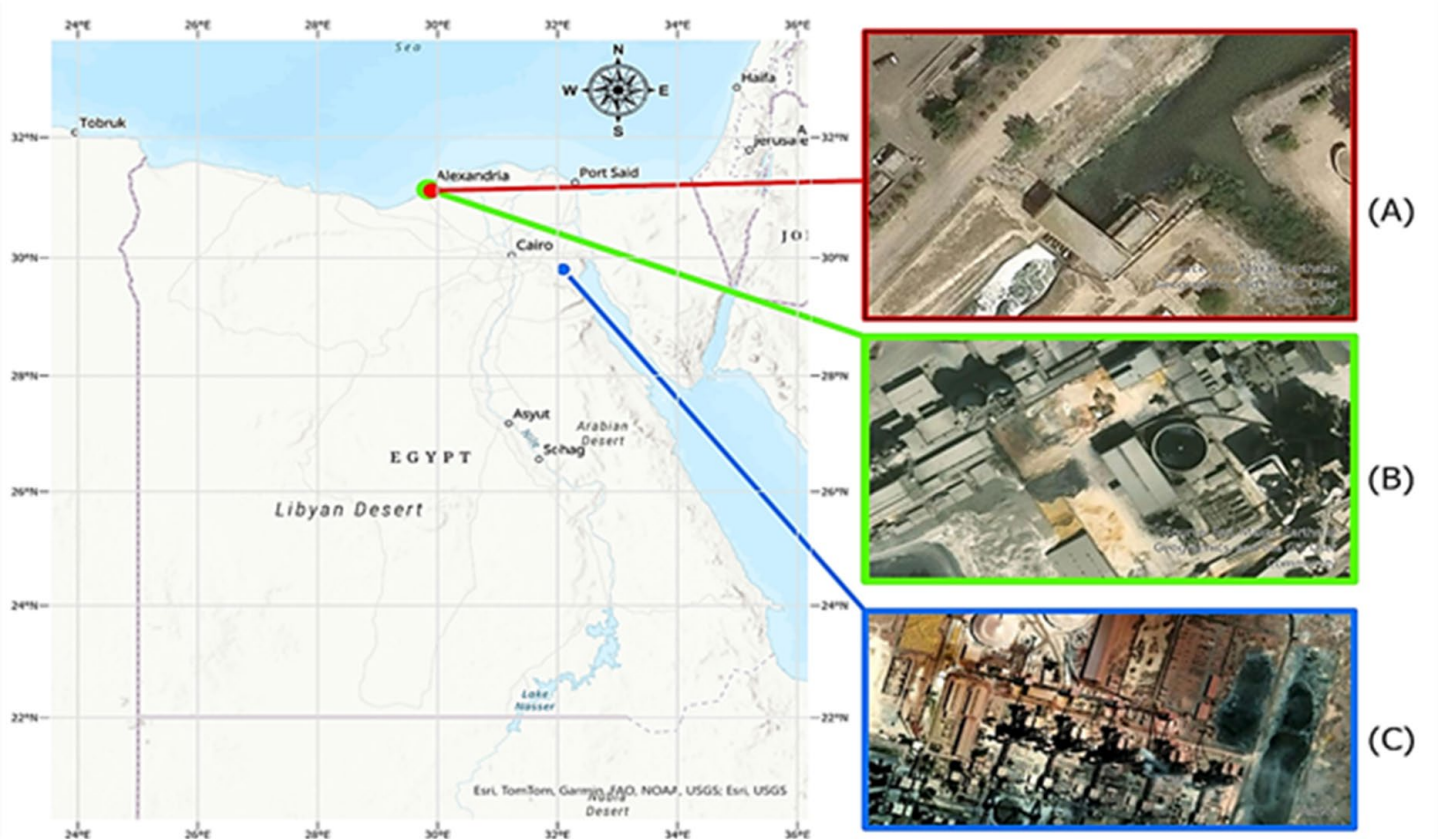
Study area for RDF and cement factories; (**A**) Nahdet Misr for Modern Environmental Services Company, (**B**) Titan Cement Plant, and (**C**) Lafarge Attaka Cement Plant.

This paper has evaluated the environmental emissions of the RDF production and based on Nahdet Misr that specializes in modern environmental services. The RDF production plant which was evaluated in this study located in Governorate of Alexandria (31.18793^o^ N, 30.00761^o^ E) (Fig. 1A). Alexandria Governorate is urban center of great significance in the Mediterranean Sea in northern part of Egypt and is considered a major center of MSW generation and treatment. In 2011 the firm has operated and managed a large part of the integrated waste management system in Alexandria which comprise of waste collection, recycling and transportation to transfer points and sanitary landfills. In the recent past, its recycling plants, especially the Montaza plant, have improved its RDF production, which has facilitated the diversion of waste materials in landfills and the adoption of the recycling aspect of waste as part of the Egyptian Environmental Affairs Agency.

Another aim of this study was to evaluate the environmental emissions of the cement production in Egypt, wherein data were gathered regarding two large Egyptian companies namely Lafarge and Titan. Titan Cement Egypt is situated at Alexandria, Egypt (31.18793°N, 30.00761°E) (Fig. 1B). Lafarge Cement Egypt is based in Ain El-Sokhna, Suez Governorate, Egypt (29.8046°N, 32.0893°E) and has one of the largest integrated cement plants in the Middle East and North African region consisting of five production lines with a total production capacity of about 10.2 million ton y^− 1^ (Fig. 1C). The two companies have undertaken the utilization of alternative fuels thus can be considered as a good case study in testing the co-processing of RDF in the cement industry.

The geographic distribution of the study locations was mapped using the geographic information system (GIS) software ArcGIS Pro 3.0.2^27^. A base map of Egypt was generated using global geographic layers, and the coordinates of the investigated facilities were imported into the GIS environment. High-resolution satellite imagery obtained from Google Earth was used to illustrate the specific locations of the facilities. The final map layout, including geographic grid, directional compass, and connection lines between the main map and the satellite images (A–C), was prepared within the GIS platform.

The spatial layout was produced by importing the geographic coordinates of the study sites into the GIS platform and overlaying them on the base map of Egypt. Directional lines were then drawn to link the main map with the corresponding high-resolution satellite images illustrating each investigated facility (A–C). The satellite panels were clipped and framed, and the final figure layout—including annotations, compass direction, and geographic grid—was prepared using standard cartographic tools to enhance spatial interpretation of the study locations.

### Production of RDF from MSW

**Fig. 2:**
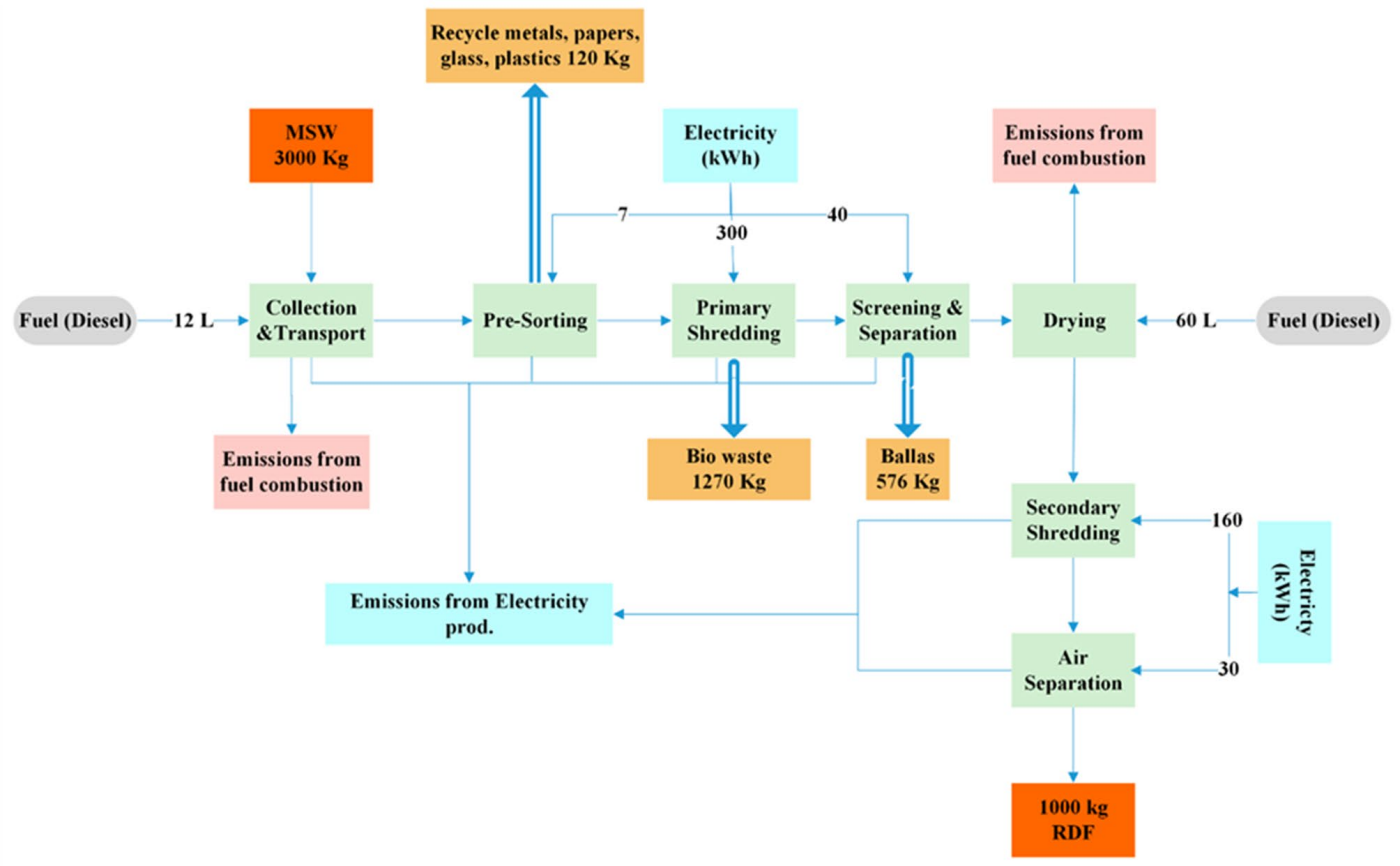
Flowchart of RDF production system.

The process of RDF production is provided in the form of a flowchart of mass and energy flows between consecutive treatment processes (Fig. 2). A collection and transportation process of MSW takes place to the facility whereby pre-sorting is done to remove bulky, inert and recyclable materials. The rest is subjected to primary shredding, screening and separation to remove fines and non-.

combustibles. Drying is used to limit moisture content and enhance calorific value after which secondary shredding is used to give a uniform particle size capable of co-processing in cement kiln. The last air separation process eliminates any light contamination impurities to produce a stable RDF product. To determine the overall mass and energy balance of the system, mass throughput, losses and energy consumption are measured at each stage.

#### MSW collection, pre-sorting, and primary shredding

The process takes off with collection and transport of approximately 3000 kg of MSW into the RDF processing plant to produce 1 ton RDF. This activity requires about 12 L of diesel fuel thus leading to emission of fuel combustion. Some might argue with me that emissions associated with transportation are also considered to be an important factor in LCA waste management systems. Once the waste reaches the facility it is subjected to a pre-sorting process in an attempt to obtain the recyclable material. This stage consumes 7 kWh of electricity and 120 kg of materials are reclaimed including metals, paper, glass and plastics. At this stage, two important purposes are accomplished: i) it reduces the amount of waste reaching the RDF production line, and II) enhances the overall sustainability of the system through recycling valuable substances, reducing the amount of virgin raw materials required and minimizing the impact associated with it^28^.

The resulting waste is then primary shredded to make the secondary shredding of the waste possible. This stage consumes about 300 kWh of electricity. It is at this stage that approximately 1270 kg of bio-waste is separated. This organic part could be channeled to other recovery mechanisms like composting or anaerobic digestion to yield biogas producing part of the integrated waste management^29^. Table (2) displays all equations utilized in the calculation of RDF production at all the processing operations of LCA.

#### Screening, drying, secondary shredding, and air separation

After the first shredding process, the product goes through a thorough screening and sorting process that aims at separating recyclable waste fractions based on size and density. This process burns about 40 kWh of electricity. Another interesting product of this step is the separation of inert materials- commonly known as ballast- that make about 576 kg. These inert fractions are not suitable to Re-Generated Dried Food (RDF) production, and thus their removal increases the fuel quality of the residual waste stream as it was shown in the recent study by Peng, An^30^. This filtered content is then taken onto the drying stage, which is an important phase to dry the material before the war faced by moisture. At this phase, the fuel used is close to 60 L of diesel fuel, which produces other combustion related emissions. Since moisture content significantly reduces the net calorific value of RDF, drying process is an irreplaceable stage of making RDF a feasible alternative fuel.

Through proper minimization of moisture level, the overall energy consumption, as well as, the combustion behavior of RDF, is significantly increased^31^. After the drying process, a secondary shredding process is done on the waste; this process tries to give the waste the size of a smaller and more homogenous particles. This secondary shredding takes about 160KWh of electricity to produce one ton of RDF. RDF particle homogeneity is the key attribute needed to get them fed successfully into cement kilns or any other industrial furnace wherein good combustion conditions are needed (Table 2). The next step is air separation to get rid of the unwanted light or fine materials like dust and very light particles, which consume about 30 kWh. By avoiding these unwanted elements, air separation increases the chemical composition and calorific value of the final RDF produced.

**Table 2:** The mass and energy balance for producing 1 ton of RDF.

Stage/Category	Key Equations	Symbol	Reference
Collection and Transport	${M}_{in}={M}_{out}=3000kg$ ${E}_{diesel}={V}_{diesel}\times{CV}_{diesel}$ ${CO}_{2}\mathrm{E}\mathrm{m}\mathrm{i}\mathrm{s}\mathrm{s}\mathrm{i}\mathrm{o}\mathrm{n}\mathrm{s}={V}_{diesel}\times{EF}_{diesel}$	Min represents the input mass (kg), M_out_ denotes the output mass (kg), E_diesel_ indicates the diesel energy (MJ), V_diesel_ refers to the diesel volume (L), CV_diesel_ signifies the diesel caloric value (MJ/L), EF_diesel_ is the emission factor for diesel combustion (MJ) equal to 2.6–2.7 kg CO_2_ L^− 1^, emission factor for diesel production ~ 0.56–0.68 kg CO₂-eq per liter of diesel, and electricity emission Factor (Grid Electricity) equal 0.647 kg CO₂-eq per kWh.	^32^ ^30^
Pre-Sorting	${M}_{in}={M}_{rec}+{M}_{rdf}+{M}_{loss}$ ${M}_{RDF}={M}_{in}-({M}_{rec}+{M}_{loss})$ ${E}_{RE}=\frac{{M}_{rec}}{{M}_{in}}\times100$	Min is the input mass (kg), Mout is the output mass (kg), M_rec_ is the mass of recyclable fractions (kg), M_loss_ is the mass lost, MRDF is the mass of refuse-derived fuel (kg), and E_RE_ represents the recycling efficiency (%).	^28^
Primary Shredding	${M}_{in}={M}_{rec}+{M}_{RDF}+{M}_{loss}$ ${M}_{RDF}={M}_{in}-({M}_{rec}+{M}_{loss})$ ${E}_{rec}=\frac{{M}_{rec}}{{M}_{in}}X100$ ${E}_{total}={E}_{main}+{E}_{OUR}+7$ ${E}_{specific}=\frac{{E}_{total}}{{M}_{in}}$ $=\frac{307}{2.85}=108kWh{ton}^{-1}$	M_IN_ represents the input mass (ton), E_rec_ denotes the recycling efficiency (%), M_rec_ indicates the mass recovered (kg), M_RDF_ refers to the RDF output mass (kg), M_loss_ signifies the mass loss (kg), E_main_ is the main shredding energy (kWh), E_total_ represents the total energy (kWh), and E_specific_ is the specific energy (kWh ton^− 1^).	^29^
Screening and Separation	${M}_{in}={M}_{rec}+{M}_{RDF}+{M}_{loss}$ ${M}_{RDF}={M}_{in}-\left({M}_{in}+{M}_{loss}\right)$ $=1550-\left(576+20\right)=954kg$ ${E}_{total}=40kwh$ ${E}_{specific}=\frac{40}{1.55}=25.8kWh{ton}^{-1}$		^30^
Drying	${M}_{in}={M}_{dry}+{M}_{evap}$ $\mathrm{a}\mathrm{s}\mathrm{s}\mathrm{u}\mathrm{m}\mathrm{i}\mathrm{n}\mathrm{g}10\mathrm{\%}\mathrm{o}\mathrm{f}\mathrm{w}\mathrm{a}\mathrm{t}\mathrm{e}\mathrm{r}\mathrm{r}\mathrm{e}\mathrm{m}\mathrm{o}\mathrm{v}\mathrm{e}\mathrm{d}:$ ${M}_{evap}=0.1X954=95.4kg$ ${M}_{dry}=954-95.4=858.6kg$ ${E}_{diesel}={V}_{diesel}XC{V}_{diesel}$ $=60X35.8=2148MJ$ ${CO}_{2}=60X2.68=160.8kg{CO}_{2}$	M_dry_ represents the mass of the dried material (kg), M_evap_ indicates the mass of evaporated water (kg), and CO_2_ refers to the carbon dioxide emissions (kg CO_2_).	^31^
Secondary Shredding	${M}_{in}={M}_{out}=858.6kg$ ${E}_{total}=160kWh$ ${E}_{specific}=\frac{160}{0.86}=186kWh{ton}^{-1}$		
Air Separation	${M}_{in}={M}_{out}+{M}_{RDF}$ Assume ${\mathrm{M}}_{\mathrm{d}\mathrm{u}\mathrm{s}\mathrm{t}}$= 50 kg ${M}_{RDF}={858.6}_{50}=808.6kWh$ ${E}_{total}=30kWh$ ${E}_{specific}=\frac{30}{0.86}=34.9kWh{ton}^{-1}$		
Final Product	${M}_{RDFfinal}=1000kg$		

Table 3 shows the chemical analysis of the RDF. Ultimately, of the 3000 kg of MSW, RDF can be produced. 1000 kg (33.3%). The RDF manufactured contained paper and cardboard at about 13.0%, plastic at 60.0%, and textiles at 15.0%, garden waste at 5.0%, and wood products at 22.0%. RDF serves as a substitute for coal in energy-intensive industries because of its high calorific value, the natural gas and hard coal fuels consumption factors equal ~ 56.1 kg CO₂ GJ^− 1^ and ~ 94.6 kg CO₂ GJ^− 1^, respectively, while the production emissions factor equal ~ 15–25 kg CO₂e GJ^− 1^ and ~ 25–35 kg CO₂e GJ^− 1^ for both natural gas and hard coal fuels, respectively. The GWP100a value for production of 1 ton RDF equal to 285.83 kg CO₂-eq, so it is very close to IPCC 2006 guidelines, suggesting it was derived correctly using the same approach, while the GWP100a value for RDF production ≈ 286 kg CO₂-eq ton^− 1^ RDF according to IPCC 2006 guidelines. This is consistent with IPCC 2006 methodology through using the official GWP factors (CO₂=1, CH₄=25, N₂O = 298). The benefits of producing RDF includes; (i) less fossil fuel consumed, (ii) greenhouse gas emissions reduced, (iii) diverted from landfills (iv) principles of the circular economy supported.

**Table 3:** The chemical analysis of the RDF.

Chemical parameter	Refused derived fuel
Carbon (%)	45.88 *±* 0.92
Hydrogen (%)	5.93 *±* 0.12
Nitrogen (%)	1.11 *±* 0.02
Sulphur	0.21 *±* 0.004
Chlorine	1.00 *±* 0.02
Mercury	0.10 *±* 0.002
Cadmium	0.70 *±* 0.61
Antimony	1.00 *±* 0.02
Arsenic	4.00 *±* 0.08
Cobalt	1.00 *±* 0.02
Manganese	100.00 *±* 2.00
Nickel	4.00 *±* 0.08
Lead	16.00 *±* 0.32
Copper	30.00 *±* 0.60
Vanadium	4.00 *±* 0.08

Table 4 shows the sensitive characteristics associated with the energy produced using RDF. Fixed carbon (FC) is calculated from proximate analysis using Eq. (1)^33^:

1 $$FC\left(\%\right)\hspace{0.17em}=\hspace{0.17em}100-(Moisture\hspace{0.17em}+\hspace{0.17em}Ash\hspace{0.17em}+\hspace{0.17em}VolatileMatter)$$

However, the calculation depends on the basis of reporting (as-received, dry basis, or dry ash-free basis). Typically, volatile matter and ash are reported on a dry basis in RDF characterization. If we assume that ash with 25.9%, and Volatile Matter 64.2% (Both on dry basis), where the fixed carbon ≈ 9.9% (dry basis). This is relatively low, which is typical for RDF dominated by plastics and paper fractions.

The high volatile matter (64.2%) indicates; high combustion reactivity, rapid ignition, strong flame propagation, good suitability for rotary kiln co-processing. High volatile matter is typical of RDF rich in plastics and cellulosic materials. The low fixed carbon (~ 9.9%) carbon means limited char formation, shorter residence time of solid carbon, faster combustion dynamics, and lower risk of unburned carbon residues compared to coal (which typically contains 45–60% fixed carbon), RDF behaves more like a highly reactive fuel rather than a slow-burning solid fuel. The energy perspective summary is volatile matter 64.2% (very high reactivity), fixed carbon ~ 9.9% (low char formation), ash 25.9% (significant mineral residue), and moisture 23.5% (reduces effective energy). In cement production, a fuel with a low fixed carbon content (around 9.9%) that leads to low char formation is generally considered an advantage, provided the fuel has a high volatile matter content.

**Table 4:** Sensitive characteristics associated with the energy produced using RDF.

Energy related items	Unit	RDF
Net calorific value	Kcal Kg^−1^	3245.0 ± 65.0
Gross calorific	Kcal Kg^−1^	3624.0 ± 72.0
Moisture at 40 °C	%	NA
Moisture at 105 °C	%	23.5 ± 0.50
Bulk density	Kg m^−3^	72.2 ± 1.40
Ashes	%	25.9 ± 0.50
Volatile matter	%	64.2 ± 1.30

### Cement production

**Fig. 3:**
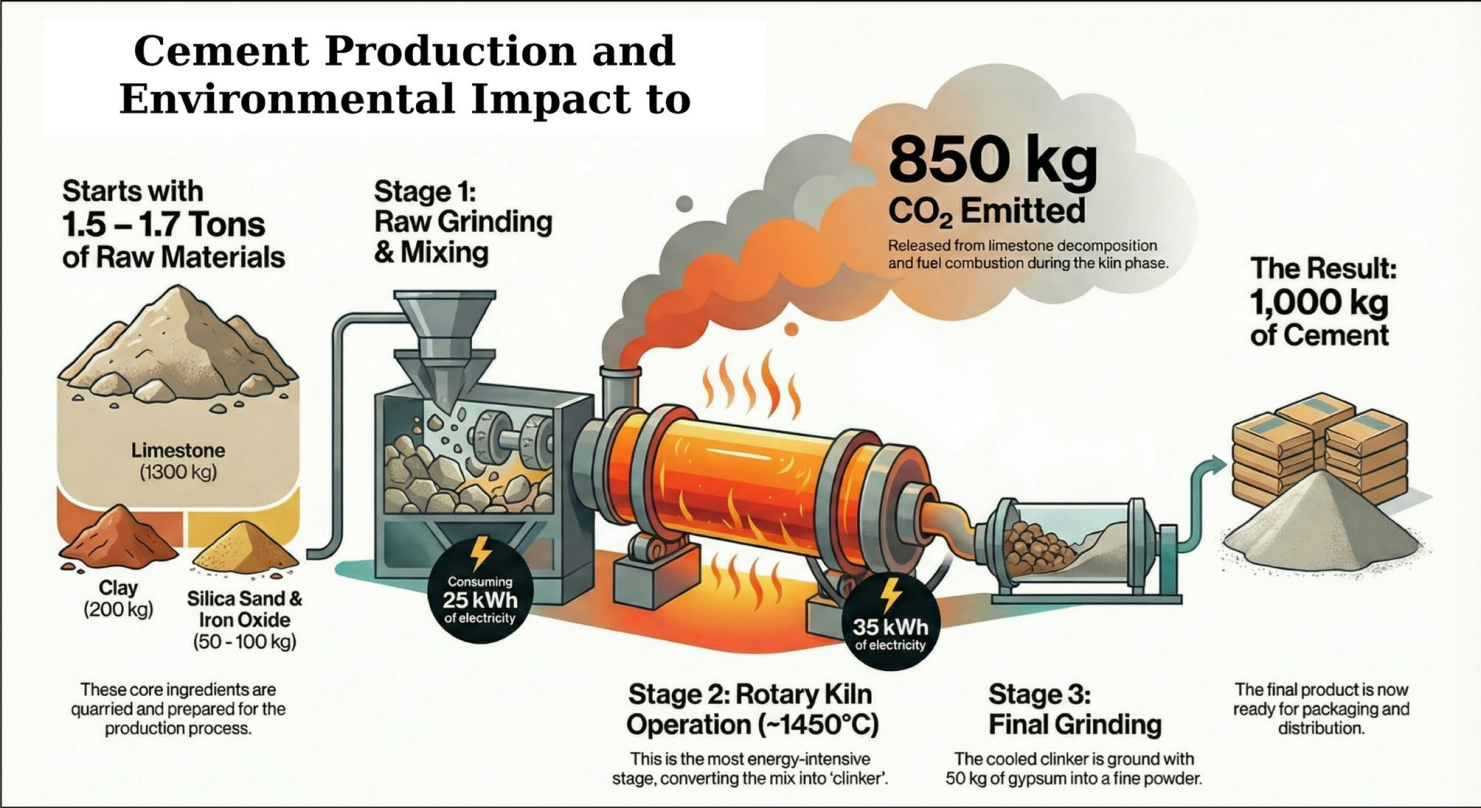
Raw materials and energy requirements to produce one ton of cement (The image was generated using Google NotebookLM Infographic based on manuscript input sources https://notebooklm.google.com/notebook/6a2bb96e-7026-40c3-a46f-7c11913e161b).

Cement production has been broken down into a process flowchart, demonstrating material and energy inputs for 1 ton, as seen in Fig. 3. It includes all 5 main production stages, such as the extraction and preparation of raw materials; raw milling and homogenization; pyro-processing; clinker cooling; and final grinding of cement. Both conventional fuels and RDF substitution are considered, along with the associated process emissions. This representation enables a consistent assessment of energy use and environmental releases across the evaluated scenarios.

#### Raw material extraction, milling, and homogenization

The cement production process consists of various stages and activities that consume significant amounts of energy and raw materials. Energy and materials are also needed to manage the environmental aspects of cement operations. Furthermore, the cement industry is the most significant producer of carbon oxides. The production of 1 ton of cement requires the following amounts of raw materials 1.5 ton to 1.7 ton of raw materials. 1300 kg limestone, 200 kg clay, 20 to 50 kg silica sand, 30 to 50 kg iron oxide. The production of cement, limestone (CaCO_3_) is the principal raw material. It is 75–80% of the raw mix. It is also the main contributor to calcium oxide (CaO) that is present in the clinker minerals. Limestone is mined from quarries using blasting and drilling, and is then moved to the crushers. The crushers break the limestone into necessary sizes. Clay or shale is the second is also necessary ingredient. It contains SiO_2_, Al_2_O_3_, and Fe_2_O_3_. These oxides are important for clinker-phase to form. These oxides are crucial for the formation of the different phases of clinker during the intense heat of the kiln. Other correcting materials such as sand (which contains silica) and iron ore are added in lesser quantities. The chemical composition of the raw materials is called the raw mix. The proportions of the materials are controlled to meet the desired values for the Lime Saturation Factor (LSF), Silica Ratio (SR) and the Alumina Ratio (AR). These ratios have a significant impact on kiln performance, energy usage, and clinker quality^34^.

The raw materials are then finely ground in vertical roller or ball mills, making raw meal in the process. Effective grinding produces a raw meal of appropriate fineness and consistency, which is important for enabling the necessary chemical reactions in pyroprocessing. Moisture can be reduced in the mill itself or removed using hot gases from the kiln. The raw meal is then ground and stored in homogenization silos, where air-blending or mechanical distribution systems are used to achieve complete chemical uniformity. The importance of the homogenization process cannot be overstated. Even small compositional differences can create problems during kiln operation, erratic fuel burn, and/or undesirable clinker mineralogy^35^. The Grinding and Mixing process used 25 kWh.

#### Pyro-processing, clinker cooling, and final grinding

The unprocessed meal is introduced into a multi-stage cyclone preheater tower, where it is heated by hot exhaust gases from the rotary kiln. This heat exchange significantly improves energy efficiency. As the temperature rises to approximately 850 to 900 °C, the raw meal undergoes calcination, during which calcium carbonate decomposes into calcium oxide (CaO) and releases CO₂. This stage is responsible for 60 to 65% of the total CO₂ emissions associated with cement production. After calcination, the material is transferred to the rotary kiln, a slightly inclined, rotating steel cylinder lined with refractory bricks. At temperatures of 1400 to 1450 °C, the material partially melts and forms new crystalline compounds. The principal minerals in the resulting clinker are tri-calcium silicate (Ca_3_SiO_5_), which provides early strength; tri-calcium aluminate (Ca_3_Al_2_O_6_), which determines setting characteristics; and tetra-calcium aluminoferrite (4CaO.Al_2_O_3_.Fe_2_O_3_), which influences clinker color and minor properties. As the molten material exits the kiln, it solidifies into gray nodules known as clinker^36^.

To preserve the desired mineral phases, the clinker must be rapidly cooled, which is done in grate coolers onto which air is blown and which rapidly reduces the temperature of the material from about 1400 °C to below 150 °C, and in so doing recovers some of the heat for use in the kiln or preheater system as combustion air. Further, if the clinker is cooled rapidly, it is more readily ground, and so desirable alterations in the mineral structure are avoided^37^. The cooled clinker is transported to the cement mills where the clinker is finely ground, together with about 4–5% of gypsum to control the setting time. Depending on the type of cement being produced, further materials are often added to improve the performance of the cement or otherwise, or to reduce the amount of Portland cement needed, e.g., limestone, granulated blast furnace slag, fly ash, and pozzolanic materials. The grinding process is so inefficient that a relatively large amount of the total electrical energy is required, some 30–40 kWh ton^− 1^ of cement. The cement is stored in silos before packing into bags or shipment in bulk^38^.

#### Materials required to produce one ton of cement

This flowchart set out in figure (3), represents the energy consumption and material inputs for the production of one metric ton (1000 kg.) of Cement. It depicts the sequential stages in the process of manufacture, again showing both the sources of energy and what gets emitted to the environment:

1. Raw materials – between 1.5 and 1.7 tons are required to produce 1 ton of cement. This comprises; 1300 kg of Limestone, (CaCO_3_), 200 kg of clay and 20 to 50 kg of silica sand and precipitated iron, oxide materials. These are then combined to produce clinker, the main cement constituent;
2. The cement production process involves multiple energy-intensive stages with substantial associated greenhouse gas emissions. Raw material preparation, including grinding and homogenization of limestone, clay, and other components, requires approximately 25 kWh of electricity per ton of clinker, producing a uniform feed for thermal processing. The rotary kiln, operating at approximately 1450 °C, facilitates clinker formation through calcination (CaCO₃ → CaO + CO₂) and subsequent sintering reactions, with a thermal energy requirement of 3200–3500 MJ per ton of clinker, corresponding to approximately 70–100 kg of coal, 100–200 kg of refuse-derived fuel (RDF), or 50–60 m³ of natural gas, depending on fuel type and kiln efficiency; electricity consumption during this phase is typically 35 kWh per ton of clinker. Clinker yield is generally 0.90–0.95 ton per ton of processed raw meal, followed by the incorporation of approximately 50 kg of gypsum (CaSO₄·2 H₂O) during final cement grinding to control setting behavior. Direct CO₂ emissions from the kiln are dominated by process emissions from limestone calcination, contributing approximately 0.52–0.60 ton CO₂ ton^− 1^ of clinker, while fuel combustion emissions contribute an additional 0.20–0.35 ton CO₂ ton^− 1^, resulting in a total kiln emission range of 0.75–1.2 ton CO₂ ton^− 1^ of clinker. Consistent with IPCC Guidelines for National Greenhouse Gas Inventories (2019 Refinement), the calcination reaction represents the largest single source of CO₂, highlighting the importance of both fuel efficiency and alternative raw materials in mitigating the carbon footprint of cement production.
3. Final Grinding & Packaging phase which transforms clinker into fine cement powder and prepares it for distribution. Although energy data for this stage is not detailed in the chart, it is generally lower than that of kiln operations.
4. Finally the output of 1000 kg of cement is produced.

Figure (3) provides a succinct overview of the environmental impact of cement manufacturing, highlighting the significant energy requirements and carbon emissions linked to the rotary kiln stage. It is particularly pertinent for life cycle assessment (LCA) studies and sustainability evaluations within the construction industry.

#### RDF substitution scenarios in cement production

Five fuel substitution scenarios (Sc-I to Sc-V) were developed to evaluate the effect of RDF integration on the thermal energy supply of cement production. The total specific thermal energy demand was fixed at 3500.2 MJ ton⁻¹ cement for all scenarios to ensure comparability and to isolate the impact of fuel substitution from process efficiency variations. Lower heating values (LHVs) of 40 MJ m⁻³ for natural gas, 30 MJ kg⁻¹ for hard coal, and 17.2 MJ kg⁻¹ for RDF were used to calculate fuel quantities. In the baseline scenario (Sc-I), 44.01 m³ of natural gas and 58.00 kg of hard coal supplied the entire energy demand without RDF. Progressive substitution was then modeled by replacing fossil fuels on an energy-equivalent basis: Sc-II (20% RDF) included 41.00 kg RDF with reduced natural gas (35.00 m³) and coal (46.50 kg); Sc-III (50% RDF) required 102.00 kg RDF with 22.00 m³ natural gas and 28.86 kg coal; Sc-IV (80% RDF) involved 163.00 kg RDF with minimal fossil inputs (9.00 m³ natural gas and 11.22 kg coal); and Sc-V (100% RDF) relied entirely on 203.50 kg RDF (Table 5).

**Table 5:** Quantity and calorific value of energy sources used in the burning process for producing one ton of cement under different scenarios.

Energy source type	Calorific value	Quantity				
		Sc-I (0% RDF)	Sc-II (20% RDF)	Sc-III (50% RDF)	Sc-IV (80% RDF)	Sc-V (100% RDF)
Natural gas (m^3^)	40 (MJ m^− 3^)	44.01	35.00	22.00	9.00	0.00
Hard coal (kg)	30 (MJ kg^− 1^)	58.00	46.50	28.86	11.22	0.00
RDF (kg)	17.2 (MJ kg^− 1^)	0.00	41.00	102.00	163.00	203.50
Total MJ ton^− 1^ cement	MJ ton^− 1^ cement	3500.20	3500.20	3500.20	3500.20	3500.20

### Life cycle assessment (LCA)

The LCA was conducted in accordance with ISO 14,040 and ISO 14,044 standards. Background life cycle inventory (LCI) data were obtained from the Ecoinvent database, ensuring methodological consistency and data reliability. The system boundary was defined as cradle-to-gate, encompassing raw material extraction and preparation, fuel production, electricity generation, clinker production, and final cement grinding.

#### LCA Framework and Scope

Goal and scope describe the objective of the assessment plus system of the product and/or process, functional unit, target audience, system boundaries, assumptions, etc. It defines the jurisdiction of the assumptions made^39^. The LCA was conducted to quantifying the impacts for the RDF production and for the manufacture of cement. Two functional units were defined: (i) RDF production of 1 ton (including waste management as an ancillary service), and (ii) Cement production of 1 ton. A cradle-to-gate system boundary was applied to both systems to capture the upstream stages but exclude the use and end-of-life stages.

The RDF production system includes MSW collection and transport, pre-sorting, primary shredding, screening and separation, drying, secondary shredding, and air separation. Inputs to the system consist mainly of mixed MSW, electricity, and auxiliary energy for drying; outputs include RDF as the principal product and inert materials, metals, and fines rejected from screening divided between recycling and disposal. Mass and energy flows at each stage of processing were quantified in order to establish a complete mass and energy balance across the whole. Environmental impacts were allocated relative to RDF on the basis of physical mass flows with the same principle applied to all the other processes of RDF production, so that the processing burdens were consistently apportioned.

Preparing raw materials, producing clinker in a rotary kiln, and grinding the finished cement are all included in the cement production system. Depending on the assessed substitution scenario, energy inputs include thermal energy and electricity provided by natural gas and hard coal, natural gas, and RDF. In the baseline scenario (Sc-I), energy demand was met with 44.01 m³ of natural gas and 58.00 kg of hard coal, excluding RDF. Subsequent scenarios modeled fossil fuel substitution: Sc-II (20% RDF) used 41.00 kg of RDF, 35.00 m³ of natural gas, and 46.50 kg of coal; Sc-III (50% RDF) required 102.00 kg of RDF, 22.00 m³ of natural gas, and 28.86 kg of coal; Sc-IV (80% RDF) involved 163.00 kg of RDF with minimal fossil fuels (9.00 m³ of natural gas and 11.22 kg of coal); and Sc-V (100% RDF) relied solely on 203.50 kg of RDF. A consistent comparison of environmental performance across various fuel mixes is made possible by the system boundary ending at the cement plant gate.

#### Life cycle inventory (LCI)

**Fig. 4:**
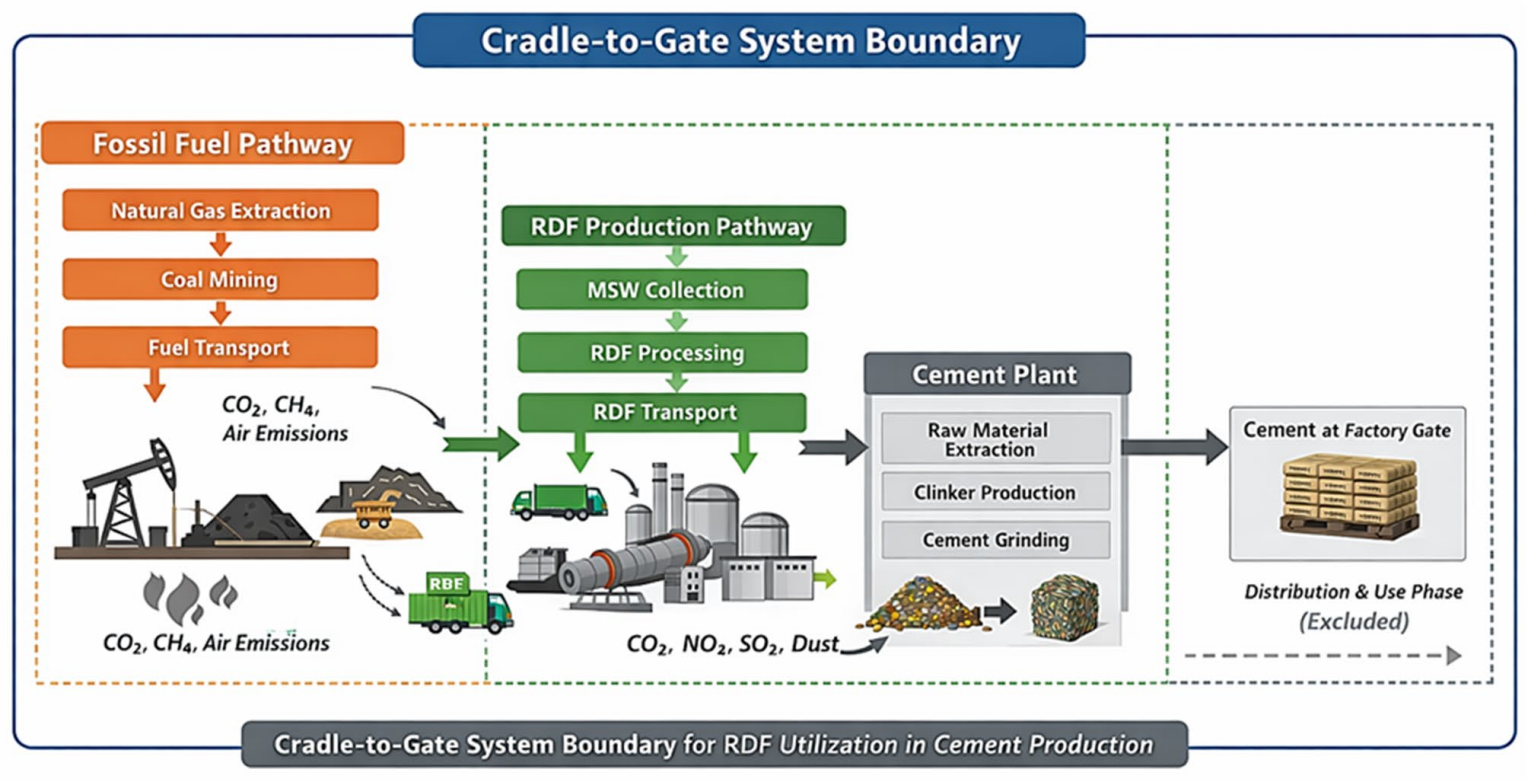
Cradle-to-Gate system boundary for LCA of RDF utilization in cement production (The image was generated using Google NotebookLM Infographic based on manuscript input sources https://notebooklm.google.com/notebook/6a2bb96e-7026-40c3-a46f-7c11913e161b).

The LCI phase entails collecting input and output inventory data that covers a variety of environmental factors in addition to the product under evaluation. Within the specified cradle-to-gate system boundaries (Fig. 4), the LCI was created to measure all material and energy inputs, outputs, and emissions related to the production of one ton of RDF (Fig. 2) and one ton of cement (Fig. 3). Primary operational data were gathered from the cement plants under investigation and the RDF production facility run by Nahdet Misr for contemporary environmental services. MSW inputs, fuel energy used for MSW collection and drying, and electricity consumption for mechanical operations (pre-sorting, primary shredding, screening, secondary shredding, and air separation) are all included in the inventory for RDF production. The final RDF product and residual fractions like metals, inert materials, and fines that are sent to recycling streams or disposal make up outputs. To ensure that the mass balance was closed, material losses and recovery at each processing stage were monitored using mass flow data.

According to the specified substitution scenarios, the LCI takes into consideration raw material inputs, electricity consumption, and thermal energy provided by natural gas, hard coal, and RDF in the production of cement. In addition to upstream emissions related to fuel extraction, processing, and transportation, direct emissions from fuel combustion and clinker production processes were also included. To guarantee uniform thermal equivalency across scenarios, energy inputs were computed using the calorific values and quantities of each fuel source.

#### Life cycle impact assessment (LCIA)

The final RDF product and residual fractions like metals, inert materials, and fines that are sent to recycling streams or disposal make up outputs. To ensure that the mass balance was closed, material losses and recovery at each processing stage were monitored using mass flow data. LCIA is a comprehensive instrument intended to evaluate possible environmental effects that align with the environmental resources noted in the LCI. In order to enable a comprehensive assessment of the product’s effects, this evaluation addresses a number of environmental issues, such as energy consumption, emissions, global warming, climate change, and water pollution^40^.

The intricate LCIA phase divides all inventory into discrete impact categories. The results of LCIA and LCI are then analyzed in the final stage. Human toxicity (HT), acidification (AC), eutrophication (EP), freshwater aquatic eco-toxicity (FE), abiotic depletion (AD), Abiotic depletion, fossil fuels (ADF), global warming potential (GWP), marine aquatic eco-toxicity (ME), and photochemical oxidation (PO) were among the environmental impacts assessed using the CML2 baseline V3.04/EU25 method. The open LCA 1.10.3 program was used to calculate these impact potentials. A thorough analysis of the environmental advantages of using more RDF in cement production is made possible by the evaluation of RDF and cement systems taken together.

#### Interpretation

The concluding phase, is an effective approach employed to assess, compute, and classify the results derived from the data provided by LCI and LCIA, while also establishing a connection by illustrating the influence each output data has on its respective impact category, ultimately defining the study’s objective^41^. During this stage, production processes and substances that have considerable impacts will be presented in a clear and comprehensive manner, followed by the formulation of appropriate recommendations.

### Economic feasibility

Egypt’s cement industry has experienced significant growth, with its production capacity increasing from 1.4% of global capacity in 2017 to 3.1% in 2022^42^. The cement industry in Egypt is a vital part of the economy, contributing 3.7% to the national GDP and consuming about 5.3% of the country’s total energy^43^.

Given the sector’s high energy intensity and reliance on imported fossil fuels, fuel substitution using RDF was evaluated as a cost-reduction strategy. Annual cement production was estimated from the rated plant capacity and utilization factor.

Egypt’s cement industry has experienced significant growth, with its production capacity increasing from 1.4% of global capacity in 2017 to 3.1% in 2022^42^. The cement industry in Egypt is a vital part of the economy, contributing 3.7% to the national GDP and consuming about 5.3% of the country’s total energy^43^.

#### Production/mass balances

2 $$AnnualCement={P}_{rated}\times\mathrm{U}$$

Where *P*_*rated*_ is the plant capacity (ton y^−1^), and U is the utilization fraction (plant availability × operating factor, dimensionless 0–1)^44^.

#### Fuel energy allocation

##### Energy supplied by RDF

3 $${E}_{RDF}=S\times{E}_{C}$$

Where *S* is the RDF thermal substitution rate (fraction), and *E*_*C*_ is the thermal energy demand per ton of cement (GJ ton^− 1^).

##### Energy supplied by coal

4 $${E}_{coal}=(1-S)\times{E}_{C}$$

#### Fuel consumption equations

##### RDF mass required

5 $${Cost}_{RDF}={E}_{RDF}\times{C}_{RDF}$$

Where *C*_*RDF*_ is the cost of RDF (including preparation & transport) ($ GJ^− 1^), and *E*_*RDF*_ is the thermal energy supplied by RDF (GJ ton^− 1^).

##### Coal fuel cost

6 $${Cost}_{coal}={E}_{coal}\times{C}_{coal}$$

Where *C*_*coal*_ is the cost of coal ($ GJ^− 1^), and *E*_*coal*_ is the thermal energy supplied by coal (GJ ton^− 1^).

##### Total fuel cost

7 $${Cost}_{fuel,tatal}={Cost}_{RDF}+{Cost}_{coal}+{Cost}_{naturalgas}$$

Where *Cost*_*fuel, total*_ is the total fuel cost using RDF + coal ($ ton^− 1^) + natural gas ($ ton^− 1^).

#### Baseline cost (coal only)

8 $${Cost}_{baseline}={E}_{C}\times{C}_{coal}$$

#### Fuel savings per ton cement

9 $${Savings}_{unit}={C}_{baseline}-{C}_{fuel,total}$$

#### Annual fuel cost

10 $$FuelCost={C}_{RDF}\times{RDF}_{mass}+{C}_{fossil}\times{Fossil}_{mass}+TipFree\_revenues$$

Where *C*_*RDF*_ ​is the delivered cost of RDF per ton ($ ton^−1^) (May be negative if you receive a tipping fee (i.e., paid to take waste). Include pre-treatment & transport costs in this unit), *RDF*_*mass*_ is the RDF mass required (ton y^−1^), *C*_*fossil*_ is the delivered cost of fossil fuel per ton (or per MJ converted consistently), *Fossil*_*mass*_ is the mass of fossil fuel required (ton y^−1^), and *TipFee_revenues* is the explicit revenue item if tipping fees are modeled separately.

#### Financial metrics

##### Fixed annualized cost (CAPEX amortized)

11 $${A}_{capex}=CAPEX\times\frac{{i(1+i)}^{n}}{{(1+i)}^{n}-1}$$

Where *CAPEX* is total capital cost ($), *n* is the project life (year), *i* is the discount rate^45^.

##### Annual operating expenses (OPEX)

12 $${\boldsymbol{O}\boldsymbol{P}\boldsymbol{E}\boldsymbol{X}}_{\boldsymbol{a}\boldsymbol{n}\boldsymbol{n}\boldsymbol{u}\boldsymbol{a}\boldsymbol{l}}={\boldsymbol{A}}_{\boldsymbol{C}\boldsymbol{a}\boldsymbol{p}\boldsymbol{e}\boldsymbol{x}}+{\boldsymbol{C}}_{\boldsymbol{f}\boldsymbol{i}\boldsymbol{x}\boldsymbol{e}\boldsymbol{d}\_\boldsymbol{o}\boldsymbol{p}\boldsymbol{s}}+{\boldsymbol{C}}_{\boldsymbol{v}\boldsymbol{a}\boldsymbol{r}\boldsymbol{i}\boldsymbol{a}\boldsymbol{b}\boldsymbol{l}\boldsymbol{e}\_\boldsymbol{o}\boldsymbol{p}\boldsymbol{s}}$$

Where *C*_*fixed_ops*_ is the fixed operating costs (labor, insurance, maintenance) ($ y^−1^). *C*_*variable_ops*_ is the variable costs ($ y^−1^).

##### Unit production cost

13 $$\boldsymbol{U}\boldsymbol{n}\boldsymbol{i}\boldsymbol{t}\boldsymbol{C}\boldsymbol{o}\boldsymbol{s}\boldsymbol{t}=\frac{{\boldsymbol{A}}_{\boldsymbol{c}\boldsymbol{a}\boldsymbol{p}\boldsymbol{e}\boldsymbol{x}}+{\boldsymbol{C}}_{\boldsymbol{f}\boldsymbol{i}\boldsymbol{x}\boldsymbol{e}\boldsymbol{d}\_\boldsymbol{o}\boldsymbol{p}\boldsymbol{s}}+{\boldsymbol{C}}_{\boldsymbol{v}\boldsymbol{a}\boldsymbol{r}\boldsymbol{i}\boldsymbol{a}\boldsymbol{b}\boldsymbol{l}\boldsymbol{e}\_\boldsymbol{o}\boldsymbol{p}\boldsymbol{s}}}{\boldsymbol{A}\boldsymbol{n}\boldsymbol{n}\boldsymbol{u}\boldsymbol{a}\boldsymbol{l}\boldsymbol{C}\boldsymbol{e}\boldsymbol{m}\boldsymbol{e}\boldsymbol{n}\boldsymbol{t}}$$

##### Revenue

14 $$\boldsymbol{R}\boldsymbol{e}\boldsymbol{v}\boldsymbol{e}\boldsymbol{n}\boldsymbol{u}\boldsymbol{e}=\boldsymbol{A}\boldsymbol{n}\boldsymbol{n}\boldsymbol{u}\boldsymbol{a}\boldsymbol{l}\boldsymbol{C}\boldsymbol{e}\boldsymbol{m}\boldsymbol{e}\boldsymbol{n}\boldsymbol{t}\times{\boldsymbol{P}}_{\boldsymbol{c}\boldsymbol{e}\boldsymbol{m}\boldsymbol{e}\boldsymbol{n}\boldsymbol{t}}+\boldsymbol{O}\boldsymbol{t}\boldsymbol{h}\boldsymbol{e}\boldsymbol{r}\boldsymbol{r}\boldsymbol{e}\boldsymbol{v}\boldsymbol{e}\boldsymbol{n}\boldsymbol{u}\boldsymbol{e}\boldsymbol{s}$$

15 $$\boldsymbol{E}\boldsymbol{B}\boldsymbol{I}\boldsymbol{T}\boldsymbol{D}\boldsymbol{A}=\boldsymbol{R}\boldsymbol{e}\boldsymbol{v}\boldsymbol{e}\boldsymbol{n}\boldsymbol{u}\boldsymbol{e}-{\boldsymbol{O}\boldsymbol{P}\boldsymbol{E}\boldsymbol{X}}_{\boldsymbol{a}\boldsymbol{n}\boldsymbol{n}\boldsymbol{u}\boldsymbol{a}\boldsymbol{l}}$$

16 $${FCF}_{t}={EBITDA}_{t}-{Depreciation}_{t}-{Taxes}_{t}-{NetCapEx}_{t}$$

17 $$\boldsymbol{N}\boldsymbol{P}\boldsymbol{V}={\int}_{\boldsymbol{t}=0}^{\boldsymbol{N}}\frac{\boldsymbol{F}\boldsymbol{C}{\boldsymbol{F}}_{\boldsymbol{t}}}{{(1+\boldsymbol{t})}^{\boldsymbol{t}}}$$

Where *P*_*cement*_ is the selling price per ton cement ($ ton^− 1^), *Other revenues* is the tip fees, sale of by-products, waste processing fees, *Depreciation*_*t*_ is the standard cash-flow line items, and *N* is the analysis horizon (year)^46^.

## Results

### Analysis of environmental impacts of RDF production

RDF is generated through the mechanical and/or biological processing of MSW, which includes sorting, shredding, drying, and separation techniques. Although RDF provides waste-to-energy advantages, its production phase has significant environmental repercussions, such as greenhouse gas (GHG) emissions, where CO₂, CH₄, and N₂O are released during preprocessing stages, air pollutant emissions including dust, particulate matter (PM), volatile organic compounds (VOCs), and odors, energy consumption characterized by high electricity requirements during processing, and residual waste generation resulting in the production of rejects (inert materials, metals, glass). In spite of these effects, RDF is advocated as a more environmentally friendly alternative to conventional waste management methods. The environmental justifications for utilizing RDF include the reduction of landfill use, substitution of fossil fuels, energy recovery from waste, a decrease in overall global warming potential (GWP), and alignment with sustainable development goals (SDG 7 (Affordable and Clean Energy), SDG 11 (Sustainable Cities), SDG 12 (Responsible Consumption and Production), and SDG 13 (Climate Action)).

Table (6) shows the environmental impact of production of 1 ton of RDF. The operations that had the highest environmental impact were in the drying process, which included an impact of 0.02679 kg C_2_H_4_ eq., then collection and transportation of MSW, which had an impact of 0.00536 kg C_2_H_4_ eq., and lastly the primary shredding which impacted 0.00438 kg C_2_H_4_ eq. in terms of environmental impact. The pre-sorting, on the other hand, was the operation with the smallest environmental impact, at 0.0001 kg C_2_H_4_ eq (Table 6).

In terms of human toxicity, primary shredding (2.95E + 01 kg 1,4-DB eq) and secondary shredding (1.57E + 01 kg 1,4-DB eq) dominate, reflecting the release of hazardous compounds during mechanical treatment, with drying also contributing substantially (1.17E + 01 kg 1,4-DB eq), whereas pre-sorting contributes the least (6.88E-01 kg 1,4-DB eq), reflecting the dominance of mechanical shredding in generating hazardous emissions (Table 6).

The global warming potential (GWP100a) is overwhelmingly driven by drying (2.11E + 02 kg CO₂ eq), while pre-sorting has the lowest impact (4.21E-01 kg CO₂ eq). This highlights drying as the most energy-intensive process. Similarly, abiotic depletion of fossil fuels is dominated by drying (2.79E + 03 kg CO_2_ eq), with pre-sorting again being the lowest contributor (2.40E + 00 kg CO_2_ eq). For abiotic depletion (kg Sb eq), primary shredding (1.70E-04 kg CO_2_ eq) shows the highest impact, while collection and transport (2.99E-06 kg CO_2_ eq) represent the lowest, suggesting resource depletion is strongly linked to shredding operations (Table 6).

In the case of the abiotic depletion (fossil fuel), the drying process has the highest depletion of fossil fuels because it has significant energy demands of 2.79E + 03 MJ, far exceeding other stages.

**Table 6:** Environmental impacts categories for production 1 ton of RDF.

Parameter	Unit	Collection & Transport	Pre-sorting	Primary shredding	Screening & Separation	Drying	Secondary shredding	Air separation		Total
Photochemical oxidation	kg C_2_H_4_ eq	5.36E-03	1.00E-04	4.38E-03	5.80E-04	2.68E-02	2.34E-03	4.40E-04	0.03999	
Human toxicity	kg 1,4-DB eq	2.33E + 00	6.88E-01	2.95E + 01	3.93E + 00	1.17E + 01	1.57E + 01	2.95E + 00	66.7662	
Global warming (GWP100a)	kg CO_2_ eq	4.23E + 01	4.21E-01	1.80E + 01	2.41E + 00	2.11E + 02	9.62E + 00	1.80E + 00	285.830	
Abiotic depletion (fossil fuels)	MJ	5.59E + 02	2.40E + 00	1.03E + 02	1.37E + 01	2.79E + 03	5.49E + 01	1.03E + 01	3537.20	
Abiotic depletion	kg Sb eq	2.99E-06	4.06E-06	1.70E-04	2.32E-05	1.50E-05	9.29E-05	1.74E-05	0.00032	
Fresh water aquatic ecotoxicity	kg 1,4-DB eq	6.09E-01	1.40E + 00	5.99E + 01	7.99E + 00	3.04E + 00	3.20E + 01	5.99E + 00	110.897	
Eutrophication	kg PO_4_ eq	7.22E-02	1.20E-03	5.16E-02	6.88E-03	3.61E-01	2.75E-02	5.16E-03	0.52534	
Marine aquatic ecotoxicity	kg 1,4-DB eq	2.09E + 03	1.10E + 03	4.71E + 04	6.28E + 03	1.04E + 04	2.51E + 04	4.71E + 03	96832.7	
Acidification	kg SO_2_ eq	3.08E-01	2.52E-03	1.08E-01	1.44E-02	1.54E + 00	5.76E-02	1.08E-02	2.04376	

The second-largest contributors are collection and transport as it represents the fuel usage during the logistics with a value of 5.59E + 02 MJ. Although the least number was found under pre-sorting, having 2.40 MJ. These findings point to high dependency of energy by the system on fossil (especially at thermal processing stages).

For abiotic depletion, primary shredding (1.70E-04 kg Sb eq) and secondary shredding (9.29E-05 kg Sb eq) are the largest contributors, while other stages remain relatively minor. Freshwater aquatic ecotoxicity is most pronounced in secondary shredding (3.20E + 01 kg Sb eq), followed by screening and separation (7.99E + 00 kg Sb eq), and collection and transport contribute the least (6.09E-01 kg 1,4-DB eq), suggesting pollutant release during mechanical separation processes.

For eutrophication, drying dominates (3.61E-01 kg 1,4-DB eq), while pre-sorting remains minimal (1.20E-03 kg PO₄ eq). The results for marine aquatic ecotoxicity are striking, with primary shredding (4.71E + 04 kg 1,4-DB eq) as the highest contributor and pre-sorting (1.10E + 03 kg 1,4-DB eq) as the lowest, underscoring the disproportionate marine toxicity potential of shredding. In the case of the eutrophication potential, the drying process is seen as the major contribution with the emission to do with muscles of energy production being 3.61E-01 kg PO₄ eq. with moderate inputs from collection (7.22E-02 kg PO₄ eq) and primary shredding (5.16E-02 kg PO₄ eq) (Table 6).

The results for marine aquatic ecotoxicity are striking, with primary shredding (4.71E + 04 kg 1,4-DB eq) and secondary shredding (2.51E + 04 kg 1,4-DB eq) dominating, highlighting the substantial marine toxicity potential of shredding operations. These values highlight the substantial marine toxicity potential of shredding operations, likely due to emissions of persistent pollutants. Finally, acidification is primarily driven by drying (1.54E + 00 kg SO₂ eq), with collection (3.08E-01 kg SO₂ eq) and primary shredding (1.08E-01 kg SO₂ eq) also contributing. Other stages contribute minimally, suggesting acidifying emissions are strongly tied to energy consumption (Table 6).

These findings underscore the need to increase the efficiency of energy conservation and switch to cleaner energy sources to mitigate the effects of acidification. Overall, the findings demonstrate that drying is the critical hotspot for global warming, fossil fuel depletion, and acidification, whereas shredding operations dominate toxicity and ecotoxicity categories. These results emphasize the need for targeted mitigation strategies in drying and shredding stages to reduce the environmental footprint of RDF production.

### The share of individual operations in the overall environmental impacts of RDF production

**Fig. 5:**
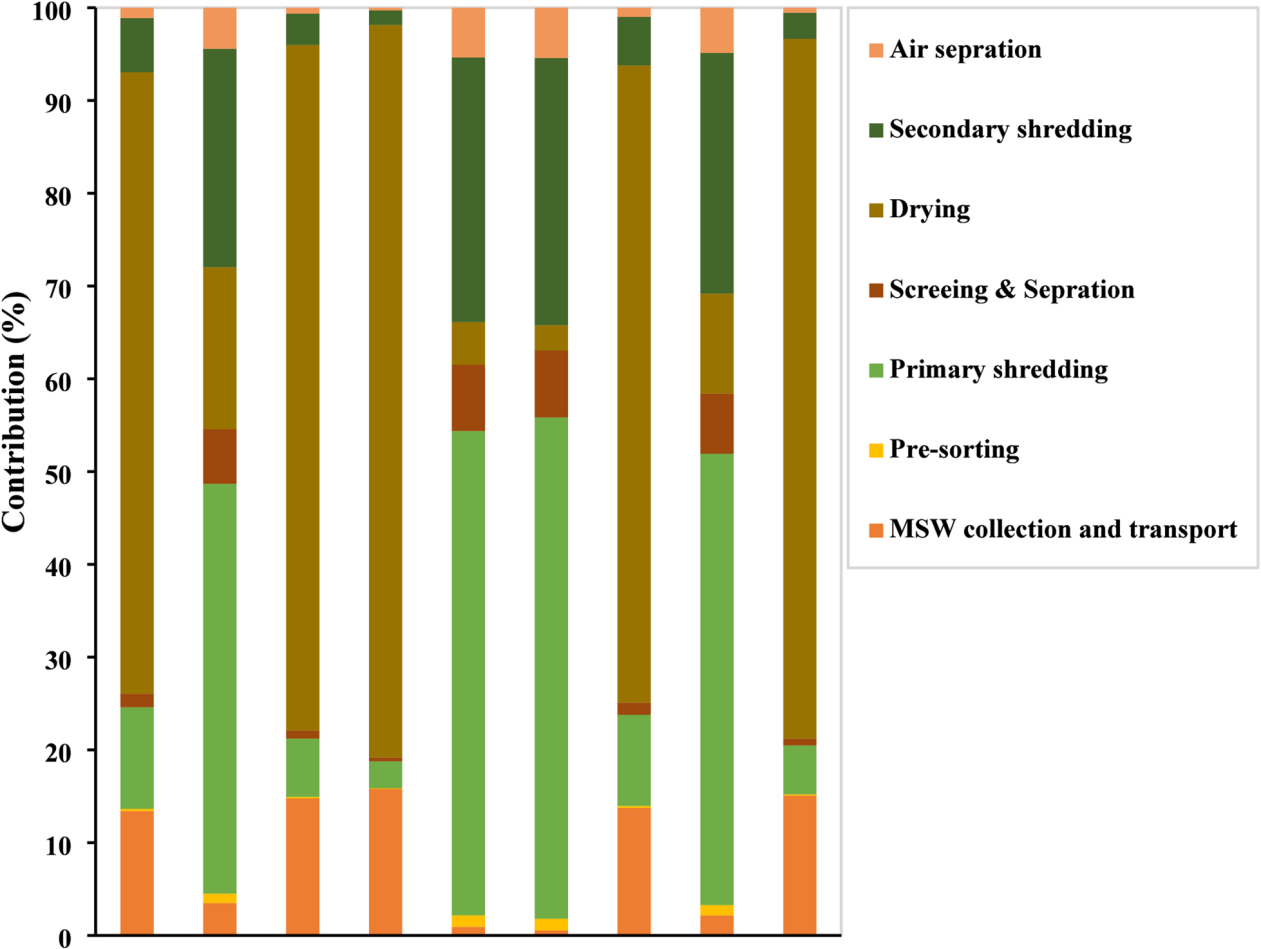
The share of RDF production operations in the environmental impact.

Figure (5) presents the relative contributions of individual RDF production operations to the overall environmental impact across multiple impact categories. The results reveal that drying is the dominant contributor across most impact categories, particularly in global warming (GWP100a), abiotic depletion (fossil fuels), and abiotic depletion, accounting for approximately 73.9%, 79%, and 75.5% of total impacts, respectively, which is primarily attributed to its intensive energy consumption. Primary shredding emerges as the leading contributor to human toxicity, Abiotic depletion, fresh water aquatic ecotoxicity, and marine aquatic ecotoxicity, ranging from 45% to 55%, reflecting significant aquatic and toxicity-related burdens generated during mechanical processing. Secondary shredding exhibits a notable contribution to Human toxicity (23.6%) and Fresh water aquatic ecotoxicity (28.8%), indicating a cumulative environmental burden across the shredding stages. MSW collection and transport demonstrates a consistent yet moderate contribution (14–16%) across photochemical oxidation, global warming (GWP100a), abiotic depletion (fossil fuels), eutrophication, and acidification, highlighting the environmental footprint of logistics operations. In contrast, air separation and pre-sorting represent the smallest contributors across all impact categories (2–5%), though pre-sorting plays a critical indirect role in reducing the environmental load on downstream processing stages. Overall, these findings suggest that optimizing energy use in the drying stage and improving effluent management in shredding operations are the most impactful strategies for reducing the overall environmental burden of the RDF production improving effluent management in shredding operations are the most impactful strategies for reducing the overall environmental burden of the RDF production.

### The proportion of distinct activities concerning the environmental effects for the RDF substitution scenarios in cement production

**Fig. 6:**
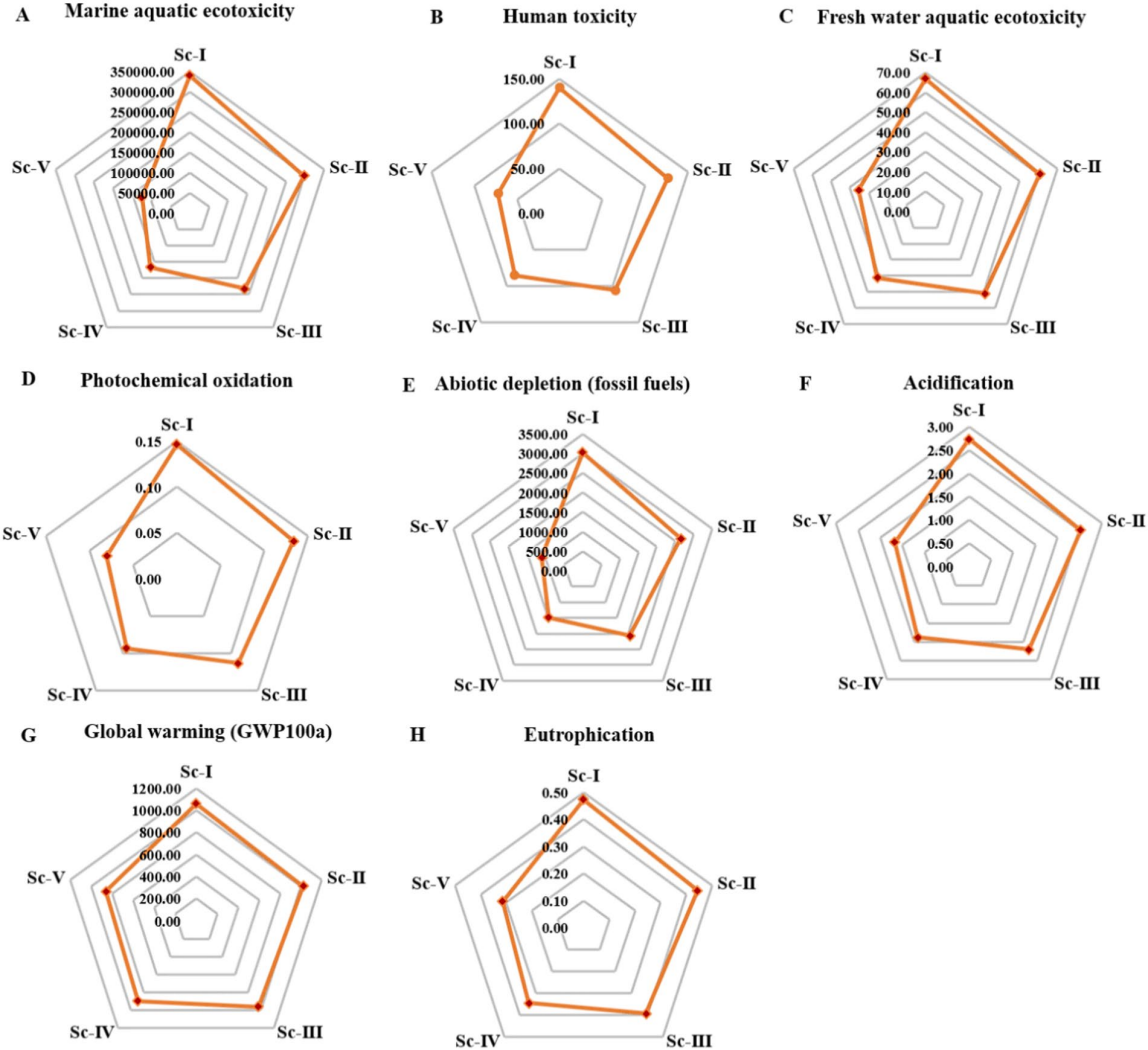
The impact of distinct activities concerning the environmental effects of cement production using RDF with different scenarios.

Figure (6) compares various scenarios of the cement production which include varying ratios of the substitution of RDF. All these worlds exhibit a decreasing trend between Sc-I and Sc-IV in all categories. This tendency implies that the higher the degree of RDF replacement, the lower the emissions, the use of fossil resources, and the amount of pollutants. This number is an effective argument in favor of the use of RDF as a more sustainable fuel option, which is closer to the environment.

Figure (6 A) shows that the marine ecotoxicity of five cement manufacturing conditions. The statistical evidence indicates that the high input scenarios of fossil fuel usually depict high values of marine ecotoxicity. Conversely, a situation where other fuels are introduced results in a significant decrease in aquatic ecotoxicity of the sea. The marine aquatic ecotoxicity data show that Sc-I was the most impactful environmental hazard of 3.40E + 05 kg 1,4-DB eq, and then was SC-II with the impact of 2.97E + 05 kg 1,4-DB eq. Sc-V, on the other hand, had the lowest marine aquatic ecotoxicity which was recorded to be 1.25E + 05 kg 1, 4-DB eq.

Figure (6B) shows how five RDF substitution conditions during make up of cement affect terrestrial ecology. Situations where fossil fuels are mostly used cause more contamination by metals and organic emissions and this was especially significant with Sc-I of 140.19 kg 1,4-DB eq. Conversely, the situations that improve utilization of RDF indicate a much smaller level of terrestrial ecotoxicity, with Sc-V reporting the minimal level of terrestrial ecotoxicity of 72.05 kg 1, 4-DB eq. These results confirm the fact that alternative fuels do have quantifiable benefits to the ecological well-being of land-based surroundings.

The eco-toxicological impacts of fresh water have been shown in figure (6 C). An increase in values means that more harmful substances, such as heavy metals and organic pollutants were released. The high fossil fuel scenarios have a higher implication because the composition of flue-gases is complex and Sc-I can be described as the most freshwater aquatic ecotoxicity at 67.03 kg 1, 4-DB eq. On the other hand, the abundance of RDF in the scenario indicates significant decline, with the higher purification nature of the combustion, with Sc-V registering the minimal freshwater aquatic ecotoxicity at 35.4 kg 1,4-DB eq.

The results of the photochemical oxidation are depicted in the figure (6D). Such situations in which levels of fossil fuel are high demonstrate increased effects particularly in the complexity of the flue-gas composition with Sc-I demonstrating the most pronounced with 0.15 kg C_2_H_4_ eq as the highest photochemical oxidation quantity. Conversely, conditions with high RDFs suggest significant cuts implying reduced cleaner burning nature with Sc-V recording the lowest photochemical oxidation of 0.08 kg C_2_H_4_ eq.

The high rates of abiotic depletion (fossil fuel) expressed in mega joules (MJ) are connected to the situation when the usage of coal and natural gas is rather intensive. On the other hand, a situation that uses RDF depicts minimized depletion of fossil fuel making the assumption of lesser effects on non-renewable sources of energy. The current observation shows the major benefit that RDF has on the preservation of natural resources as the maximum and minimum values of the abiotic depletion (fossil fuels) were registered at 3032.69 MJ and 1109.74 MJ under Sc-I and Sc-V respectively (Fig. 6E).

Figure (6 F) shows the ability of the acid to fluctuate through the emission in the SO_2_-eq. The findings indicate that coal intensive situations generate significant amount of acidifying gases. A significant decreasing effect of SOx and NOx emission results with the introduction of RDF, thus reducing the effects of acidification. This underlines the possibility of RDF being an effective policy towards reduction of environmental pressures with regard to acid rain. The minimum and maximum acidification potential values were observed with Sc-V and Sc-I of 1.68 and 2.73 kg SO_2_ eq., respectively.

Abiotic depletion is an assessment of how non-renewable mineral resources (other than fossil fuels) were used against abiotic depletion. Although the average numbers are not huge, there are situations that suggest growth of demand associated with a range of processing processes. RDF lifecycle scenarios in which there are high levels of RDF show moderate levels of depletion since there is less mining. Finally, the figure shows the advantages of resource efficiency of RDF pathway, where the lowest and largest values of abiotic depletion were obtained with Sc-V and Sc-I by 3.631E-04 and 4.169E-04, respectively.

The global warming potential represented in the chart (6G) in kg CO_2_-eq. is greater in those situations where fossil fuels are largely relied on and numb with an increased usage of RDF. As evident in the illustration, the replacement of coal and natural gas with RDF has tremendous climate benefits. The emission reduction is linear, and it directly depends on the level of fuel replacement. It is worth noting that the maximal GWP100a was determined with Sc-I where it was 1060.80 kg CO_2_-eq., and the minimal GWP100a was observed with Sc-V where GWP100a was 858.19 kg CO_2_-eq.

The graph (6 H) elaborates the possibility of eutrophication, in the form of PO_4_ incongruent equivalents. High amounts of eutrophication are normally linked with the release of the NOx and ammonia. Cases, which use a smaller amount of fossil fuel and more RDF, show much less eutrophication values. The figure highlights the RDF role in reducing the emissions of nutrient loading in aquatic environments. The maximum eutrophication was observed with Sc-I at 0.47 kg PO_4_-eq., and the minimum eutrophication was observed with Sc-V at 0.31 kg PO_4_-eq.

The integrated results confirm that RDF utilization in cement kilns provides consistent environmental benefits across all evaluated impact categories. Higher substitution scenarios (Sc-IV and Sc-V) demonstrate substantial reductions in fossil resource consumption, ecotoxicity, and greenhouse gas emissions. However, further environmental improvements would require complementary mitigation strategies addressing calcination emissions and clinker factor reduction. Overall, RDF substitution represents an effective transitional pathway toward more sustainable cement production, particularly in regions with high landfill burdens and fossil fuel dependency.

### The enhancement of different scenarios comparing with Sc-I on distinct activities on environmental impacts of cement production using RDF

**Fig. 7:**
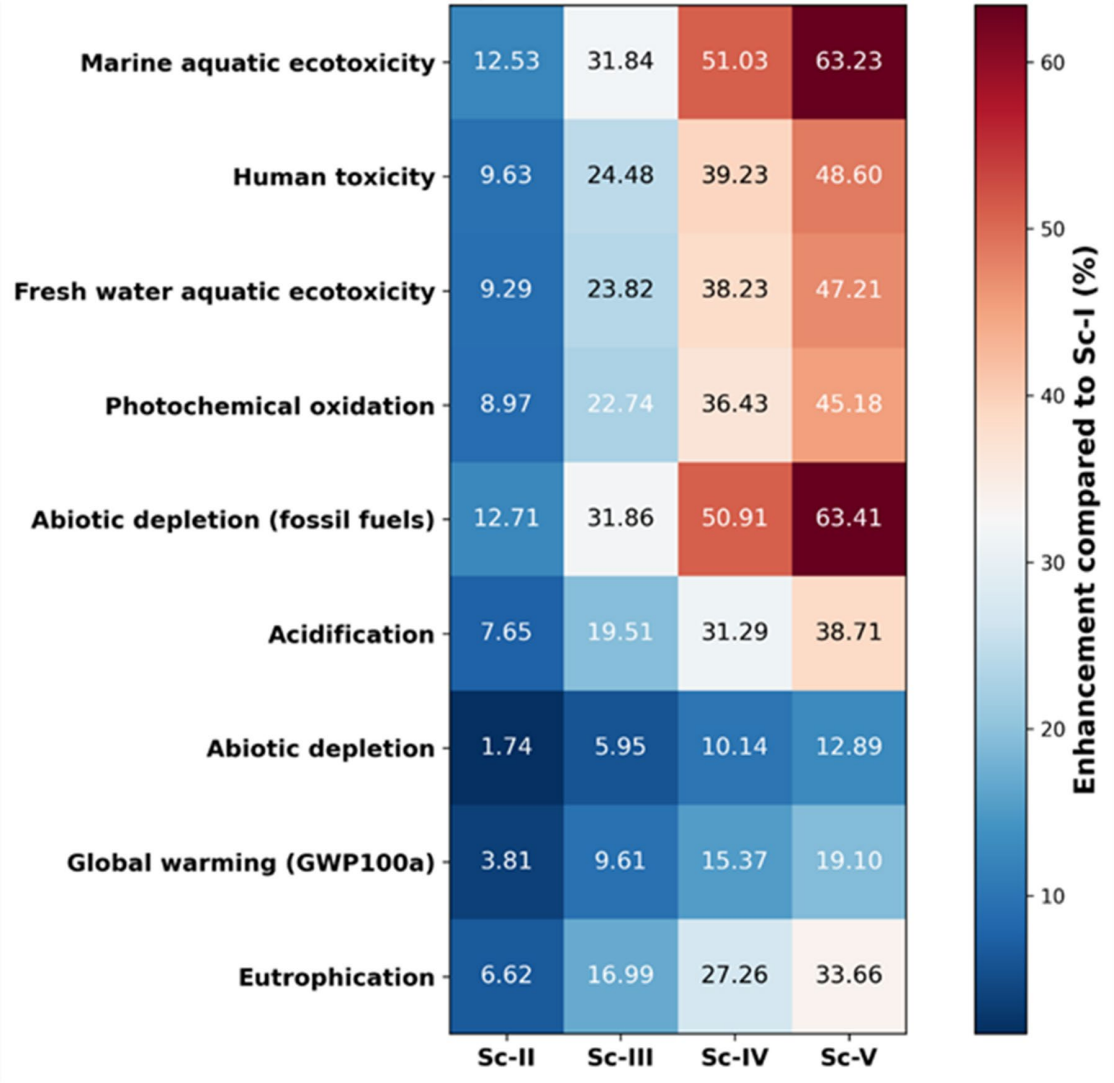
The enhancement of different scenarios compared with Sc-I on distinct activities concerning the environmental effects of cement production using RDF.

Figure (7) illustrates the percentage enhancement of environmental impact categories for Sc-II, Sc-III, Sc-IV, and Sc-V relative to Sc-I within the LCA framework of cement production incorporating RDF. A clear progressive trend is observed across all impact categories, with enhancement values consistently increasing from Sc-II to Sc-V. This pattern indicates that the incremental integration or optimization of RDF within the fuel mix systematically improves environmental performance. Sc-II shows modest improvements (generally < 13%), suggesting limited substitution or operational optimization. In contrast, Sc-III and Sc-IV demonstrate moderate improvements (approximately 20–51%), reflecting more substantial fossil fuel displacement and associated emission reductions. Sc-V exhibits the highest enhancement across all categories, reaching up to 63.41% for abiotic depletion (fossil fuels) and 63.23% for marine aquatic ecotoxicity, highlighting the strong environmental benefits achieved under the highest RDF substitution scenario.

The most pronounced improvements occur in abiotic depletion (fossil fuels) and marine aquatic ecotoxicity, followed closely by human toxicity and freshwater aquatic ecotoxicity. These results suggest that RDF substitution primarily reduces the consumption of non-renewable fossil resources and lowers pollutant emissions linked to upstream fossil fuel extraction and combustion processes. Similarly, substantial enhancement in photochemical oxidation and acidification indicates reduced emissions of NOₓ, SO₂, and volatile organic compounds associated with conventional fossil fuels.

**Fig. 8:**
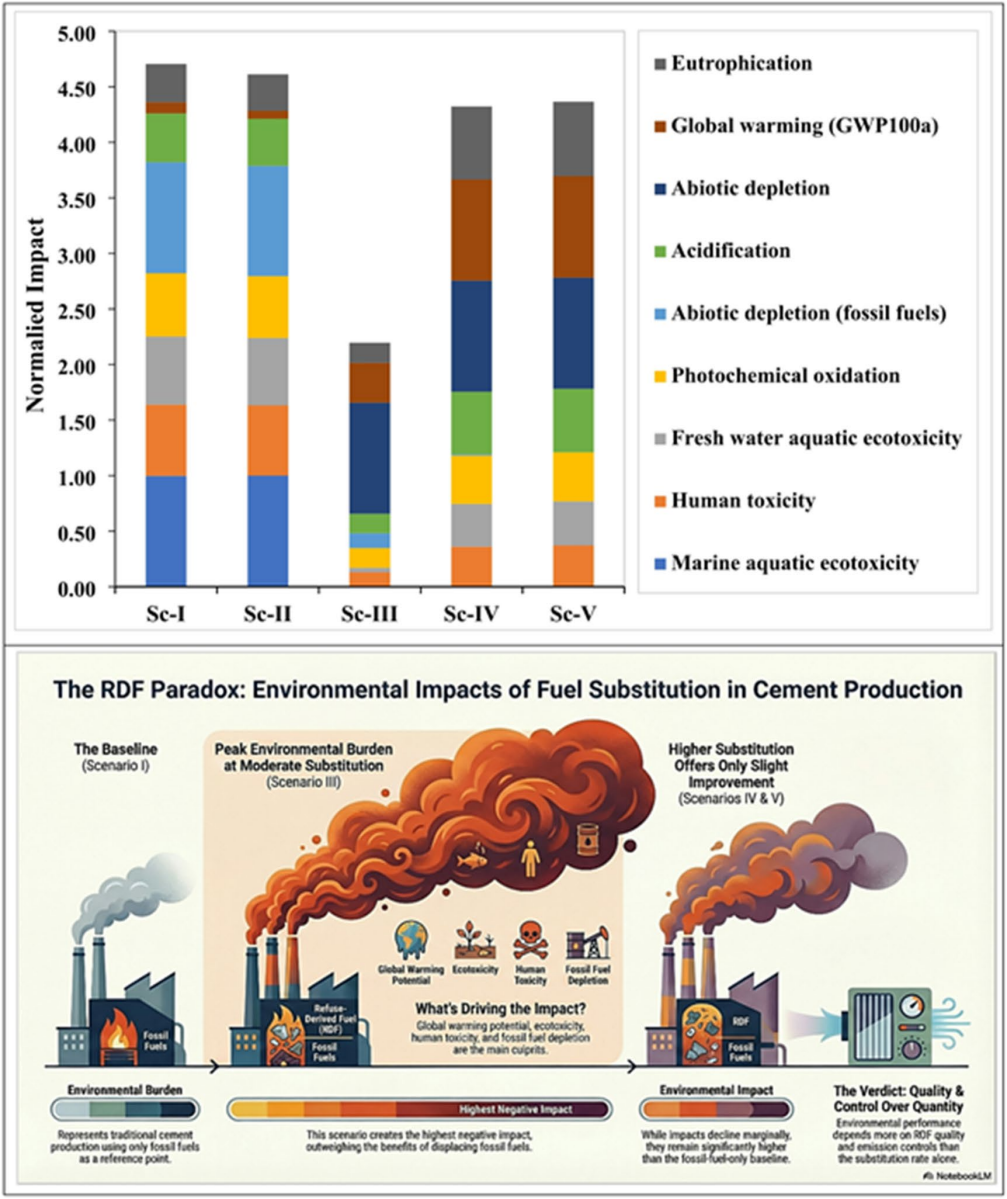
Contribution to the normalized impacts from four RDF substitution scenarios in cement production. The figure compares the normalized values of environmental-related impacts between the five RDF substitution scenarios (The second image was generated using Google NotebookLM Infographic based on manuscript input sources https://notebooklm.google.com/notebook/6a2bb96e-7026-40c3-a46f-7c11913e161b).

Global warming potential (GWP100a) shows a moderate but consistent improvement trend (3.81–19.10%), reflecting partial decarbonization of the thermal energy supply. However, the comparatively lower improvement magnitude relative to resource depletion categories suggests that process-related CO₂ emissions from limestone calcination remain a dominant contributor in cement production and are not directly mitigated by fuel substitution alone. Eutrophication and abiotic depletion (elements) display relatively smaller but steady enhancements, indicating secondary benefits associated with reduced upstream extraction and combustion emissions (Fig. 8).

Overall, the Heatmap demonstrates that increasing RDF substitution in cement kilns systematically enhances environmental performance across multiple midpoint impact categories. The progressive shift from red to blue across scenarios confirms that higher RDF integration contributes to resource conservation and emission reduction, although its influence on process-related greenhouse gas emissions is comparatively constrained. These findings support RDF utilization as an effective strategy for improving the environmental sustainability of cement manufacturing within a cradle-to-gate system boundary.

### Contribution to the normalized impacts from four RDF substitution scenarios in cement production

Figure (8), shows the Life cycle impact assessment (LCIA) normalized results of four scenarios of RDF substitute scenarios (Sc-I to Sc-V) in the cement manufacturing calculated according to the Life Cycle Assessment (LCA) scheme of ISO 14,040 and ISO 14,044 standards. This is achieved through the normalization process, which makes comparisons among different categories of impact easier since the results are represented in comparison to a common point of reference making emphasis to the relative contribution of each environmental impact in each scenario. The figure demonstrates the impact categories contribution to the normalized impacts of cement production at different levels of five RDF (Refuse-Derived Fuel) substitution case where Sc-I is the reference and Sc-II to Sc-V is the progression of RDF substitution. All RDF substitution scenarios demonstrate a significant growth in the total environmental impacts, when compared with the reference case (Sc-I). The cumulative effect is rapidly increasing in Sc-II and reaches maximum in Sc-III and thereafter it slightly decreases in Sc-IV and Sc-V, which means that greater the extent of RDF substitution, the less the environmental burdens reduce the cumulative effect of environmental factors.

The normalized impacts across Sc-II to Sc-V are predominantly caused by; Global warming potential (GWP100a), Abiotic depletion (fossil fuels), Ecotoxicity-related category (marine, freshwater and terrestrial), and Human toxicity. These categories separately represent most of the overall normalized impact in all RDF substitution cases. Impacts experienced with Sc-II were significantly higher compared with Sc-I and the highest contributors are terrestrial and freshwater ecotoxicity, global warming, and abiotic depletion, which implies that the RDF combustion emissions and other related upstream effects dominate the picture of the environmental profile. Similarly, it is observed that Sc-III had the greatest normalized impact compared to all other scenarios.

Almost all the impact categories are at the peak, especially the global warming, acidic and toxicity-related impacts. It shows that at this level of substitution, the tradeoffs of using RDF on the environment exceed the tradeoffs of displacing fossil fuel. When comparing sc-IV and Sc-III, a slight decrease in the total normalized impact is observed. Ecotoxicity and global warming contributions become smaller, meaning a higher level of fuel efficiency or enhanced control of emissions at greater substitution levels. Similarly, Sc-V was also on a downward trend but the total contribution is significant.

The contribution pattern is not much different than that of Sc-IV and global warming, and toxicity continues to prevail suggesting that the declining returns in environmental conditions are being obtained in the case that the levels of RDF replacement are very high. The change in the profile of environmental impact of cement production by RDF replacement is great. Moderate RDF substitution (Sc-III) has the greatest environmental impact primarily because of the more numerous emissions (related to climate change and toxicity groups).

Additional replacement of RDF (Sc-IV and Sc-V) leads to marginal gains, but not comprehensive compensation of the additional effects. The emission properties of the RDF combustion and fuel structure are highly related to environmental performance as opposed to the substitution rate. All in all, the findings indicate that in as much as RDF substitution is able to decrease fossil fuel dependency, the effect on the environment relies decisively on the extent of the substitution, the quality of the RDF, and the approach to controlling emissions.

### Economic feasibility analysis

#### RDF production

The utilization of RDF produced at the Nahdet Misr for MSW treatment facility requires targeted capital investments primarily related to system integration and material handling. As RDF is generated within the same industrial zone, investment needs are concentrated on reception, feeding, and environmental control systems rather than long-distance transport infrastructure. fixed costs and variable costs of the fuel production system of Nahdet Misr for modern environmental services were calculated with the calculation of profit and selling price per ton of RDF were as follows: investment costs achieved 4.123 $ ton^− 1^, while variable costs were 12.51 $ ton^− 1^ while the revenue was 3.33 $ ton^− 1^ whereas the selling price inclusive of profit and total costs was estimated at 19.963 $ ton^− 1^ (Table 7). This is largely a reasonable profit for the company as the company’s annual production is 73,000 ton y^− 1^, knowing that the Egyptian Ministry of Environment does not spend subsidies for environmentally safe factories or deduct taxes collected in 2025; Alternatively, there are incentives through financial facilities, technical support, and the adoption of clean technologies, in addition to tax exemptions proposed in investment laws (e.g. rebate of the cost of environmentally compatible machinery).

According to that if we take into account the cost saving related to that the Titan’s reliance on imported coal, which is subject to greater market fluctuations, significantly increases the economic benefits of co-processing RDF including; savings from coal reduction: 16.5–27.5 $ ton^− 1^ of cement, benefits from CO₂ reduction 8.8–16.5 $ ton^− 1^ of cement, and advantages from RDF gate-fee 16.5–33 $ ton^− 1^ of RDF. On the same context the net overall, economic advantage is projected to be between 13.2 and 58.3 $ ton^− 1^ of cement, positioning RDF substitution as one of Titan’s most economically viable strategies.

**Table 7:** Economic feasibility analysis for calculating the selling price involves total costs and revenue for producing one ton of RDF.

Category	Component	Cost ($ ton^− 1^)
**Capital Investment**	RDF Reception & Storage (Enclosed facility, reinforced flooring, ventilation)	0.823
	Material Handling Systems (Conveyors, hoppers, cranes)	0.392
	Fire Suppression Systems	0.166
	Feeding Modifications (Dosing lines, pneumatic transport, burner adjustments, control systems)	1.151
	Emission Control Enhancements (SNCR system, activated carbon injection, CEMS upgrade)	1.591
**Total Capital Investment**		**4.123**
**Operating Costs (OC)**	RDF Processing (Moisture management, screening, quality control)	5.50
	Maintenance (Conveyors, storage, and safety systems)	5.50
	Labor	1.51
**Total OC**		**12.51**
**Revenue (10%)**		**3.33**
**Total Cost + Revenue (Selling price)**		**19.963**

#### Cement production

In the same context of evaluating the economic feasibility of using RDF in cement production, the study compared the selling price per ton of cement using traditional energy sources with the selling price per ton using 20% ​​RDF with natural gas and hard coal. The results were as follows: The capital investment costs were 0.026 $ ton^− 1^ and 0.03256 $ ton^− 1^ without and with using 20% RDF respectively, on the other hand, the operating costs were 60.944 $ ton^− 1^ and 55.293 $ ton^− 1^ without and with using 20% RDF respectively, while the net revenue was 6.094 $ ton^− 1^ and 6.082 $ ton^− 1^ without and with using 20% RDF respectively, on the other hand the cost saving per 1 ton of cement was 0.134 $ ton^− 1^ for applying 20% RDF comparing with natural gas and hard coal application, which will increase with increasing the RDF sharing percentage (Table 8).

**Table 8:** Economic feasibility analysis for determining the selling price of producing one ton of cement using RDF, including the calculation of total costs and revenue.

Category	Component/Item	Cost without RDF ($ ton^− 1^)	Cost using 20% RDF ($ ton^− 1^)
**Capital Investment**	RDF Storage & Homogenization (Enclosed bunker, automated systems)	0.00	0.0066
	Mechanical Systems (Continuous feeding)	0.00308	0.00308
	Safety Systems (Fire and gas detection)	0.0011	0.0011
	Dosing & Delivery (Dosing units, pneumatic lines)	0.00616	0.00616
	Kiln/Calciner Upgrades (Burner mods, control systems)	0.00286	0.00286
	Emission Control (SCR/SNCR, activated carbon injection)	0.01166	0.01166
	Monitoring Upgrades (Continuous monitoring)	0.0011	0.0011
**Total Investment Requirement**		**0.026**	**0.03256**
**Operating Costs**	Quality Control (Engineered RDF standards)	0.00	2.5
	Maintenance (Pneumatic systems, burners, filtration)	5	5
	Coal price	5.897	4.690
	Natural gas Price	24.940	19.995
	RDF	0.000	1.230
	Clay	1.096	1.096
	Limestone	8.905	8.905
	Silica sand	1.875	4.688
	Iron oxide	0.240	0.240
	Gypsum	1.233	1.233
	Labor	8.027	6.89
**Total OC**		**60.944**	**55.293**
**Revenue (10%)**		**6.094**	**6.082**
**Total Cost + Revenue (Selling price)**		**67.039**	**66.905**

At first glance, any investor would choose to use fossil fuels in production, considering the final total profit per ton, knowing that the average production of Lafarge Cement Plant is about 10,000,000 ton y^− 1^. However, when considering the amount of environmental sustainability and the extent of support that the state will provide through reducing taxes and fees on production inputs, the situation will change radically. This is if most countries apply environmental incentives, which encourages investors to invest in these industries while applying environmentally sustainable systems.

## Discussions

### Positive effects of adding RDF in cement production (LCA Perspective)

#### Reduction in environmental impacts

The substitution of traditional fossil-based fuels with refuse-derived fuel (RDF) in cement production has been universally considered as an appropriate measure in order to mitigate the harmful effect of the environment, especially GHGs. The use of RDF in comparison with the use of conventional fuels (coal, fuel oil, and petroleum coke) always causes a decrease in CO_2_ emissions and a increase in the overall environmental performance^6,47^. Past works have established that coal can be replaced by RDF and it not only cuts greenhouse gas emission but also curbs acidification, the development of smog in summer, nitrification, the possibility of carcinogenicity, and costs associated with landfills^47,48^.

On a numerical scale, RDF application in cement kilns can lead to the reduction of CO_2_ emissions by about 3.8 ton of RDF ton^−^1 of waste and at the same time sending over 60% of waste to the landfill^47^. These benefits are evident in the current results, which showed increasing substitution of the coal and natural gas with RDF with the consequences of strong climate benefits. The decreases in emissions were in a definite direction, and were directly connected to the extent of the fuel replacement. It was shown that Sc-I (1060.79622 kg CO_2_-eq) had the highest GWP100a and Sc-V (858.185 kg CO_2_-eq) had the lowest, which demonstrates the performance of high RDF substitutes in reducing the effects of climate change.

Besides cutting down of emissions, the use of RDF increases the efficiency of energy recovery and eliminates the use of virgin natural resources without affecting cement quality. In previous studies, it was found that the total impact of the environment decreases by a maximum of 12% when fossil fuels were substituted with RDF and other alternative fuels^49–51^. In line with these studies, the current findings show that the total environmental impacts are reduced in up to 13% and significant increases are observed in climate change, human health and measures of ecosystem quality.

#### Waste management and landfill reduction

RDF incorporation into cement manufacture is also a sustainable RDF has been applied as a solution to MSW management by redirecting waste out of landfills and encouraging the recycling of materials and energy^6,52,53^. Past studies have found that there is a distinct negative correlation between the ratio of waste to be discarded to landfills and the amount of fuel displaced in cement kilns, such that RDF usage leading to the decrease in landfills was the direct consequence of its usage^53^.

The findings of the current study put a strong support on this relationship. Reduction in quantity of wastes which went to landfills corresponded with the increase in the share of RDF. Sc-V with the highest level of RDF being used per ton of cement produced had the least total impact on the environment. This situation also had a high level of energy demand reduction equivalent to reaching out to 73.33 per cent less energy that was required to produce one ton of cement. The findings provide a focus on the two-fold nature of RDF to enhance the process of waste management and decreased energy usage in the production of cement.

#### Improvement of environmental impact categories

The regular reports of environmental benefits of RDF in place of conventional fossil fuels in LCA studies are invariably positive in the reduction of acidification, eutrophication, photochemical oxidant formations, and the depletion of fossil resources^21,48,54^. Overall, the most positive environmental results are observed in case of a greater amount of RDF replacement in producing clinker^49^.

The findings of this research are clearly in line with the same pattern. Progressive enhancement of all the assessed categories of environmental impacts resulted because of the increased RDF substitution. Sc-V had the largest decreases in acidification, eutrophication, photochemical oxidants, and abiotic depletion of fossil fuels with the rate improvement of 38.71, 33.66, 45.18, and 63.41, respectively. This was succeeded by Sc-IV that had relative decreases of 31.29, 27.26, 36.43, and 50.91. Sc-II on the other hand showed minimal improvements, which were, 7.65, 6.62, 8.97 and 12.71, respectively. These results evidently show that there is a significant improvement in the environmental performance with greater level of substitution of RDF in various categories of impacts.

Other researches have reported that the application of RDF in cement production is not a major threat to the human health and the surrounding communities^55^. However, according to LCA studies, the more pronounced the content of RDF in cement manufacturing, the diminished the environmental effects of the process would resemble than in a situation where only traditional fossil fuel is used in the manufacturing of cement (e.g., petroleum coke or coal). Indicatively, the comparative results of a situation of 100% petroleum coke and a mix of 20% RDF and 80% petroleum coke were found to have a lower impact in all categories evaluated such as the abiotic resource depletion, acidification, eutrophication, the global warming, the stratospheric ozone depletion, photochemical smog, and the toxicity to the earth in case of RDF addition^56,57^.

Similarly, other areas have seen such trends whereby the addition of RDF in cement kilns also resulted in a decrease in acidification, GHG emissions, eutrophication, summer smog, landfill expenses, and even carcinogenic potentiality^19,58^. The advantages of such environmentally soundness are especially noteworthy when discussing emissions made because of the replacing the fossil fuels, whose degrees involve the range between 2% and about 23, depending on the case scenario^52^. These observations are supported by the results of the current study: human toxicity reduction in the case of Sc-II, Sc-III, Sc-IV, and Sc-V was observed, respectively as 9.63%, 24.48, 39.23 and 48.60 per cent compared to the case of Sc-I, which indicates zero use of RDF. Besides that, the rising critical percentage of RDF in the production of cement also led to the decrease in acidification, GHG emissions, eutrophication, landfill expenses, and possible carcinogenic hazard, which once again asserted the multiple environmental and health benefits of RDF incorporation.

### Negative effects and potential risks of RDF

#### Air emissions and pollutant generation

Although this process has advantages in terms of environmental conservation, such utilization of RDF in pre-calciner processes may result in the release of pollutants, such as nitrogen oxides (NOx), sulfur oxides (SOx), heavy metals and dioxin which could be harmful to human health and the environment^59^. Even in case of alternative fuels like RDF, the rotary kiln emissions are the largest cause of environmental impacts^51,60^.

The concentration of chlorine in RDF is a special issue of concern, which is capable of causing corrosion through the vaporization and condensation of alkali chlorides, leading to higher dioxin and furan emissions, depending on the operating conditions^55,61^. This experiment produced about 750 kg of CO^2^ per ton by a clinker rotary kiln with a temperature of 1450 °C. RDF chemical analysis showed that the content was chlorine 1.00±0.02, mercury 0.10 ± 0.002, cadmium 0.70 ± 0.61, antimony 1.00 ± 0.02, arsenic 4.00±0.08, cobalt 1.00 ± 0.02, manganese 100.00 ± 0.20, nickel 4.00±0.08, lead 16.00 ± 0.32, copper. The greatest concentrations were noted in the form of manganese and copper, lead and arsenic and vanadium and nickel and so the need to have effective emission monitoring and control is significant.

### Operational and technical challenges

The ash level in RDF is quite high with considerable portions of alkali and alkaline earth metals that may intensify particulate matter (PM) emission and alleviate fouling, slagging, and corrosion in the boiler heat exchanger. Such effects could damage efficiency and safety in the operation^61^. The air emissions should thus be regulated with care to make sure that the gains of environment using RDF in its place are achieved to the maximum extent^62^.

The given paper tested several cases with different ratios of RDF to fossil fuel. Findings show that the negative environmental effects reduced in a downward trend as the levels of RDF in the shares increased, with Sc-V having the best results regarding environmental and human health indicators. The findings are in line with the general aim of reducing the pollution caused by the cement industry by implementing a strategic use of alternative sources of fuel thus enhancing a production process that is environmentally friendly.

### Transportation and system boundary considerations

A number of works have emphasized that emissions associated with the movement of RDF and other renewable fuels may greatly affect the total environmental impact, at times more significant than the emissions of fuel combustion per se^50,51^. MSW collection and transport made contributions of 13.4% and 15.8% and 13.7% to the process of photochemical oxidation, abiotic depletion of fossil fuels, eutrophication and acidification respectively, hence the need to incorporate the transport-based emissions contributed by MSW.

### Economic feasibility and cost-benefit analysis

The RDF use in the cement production industry is environmentally and economically beneficial in comparison with landfill waste or incineration, especially when mixed textiles and MSW are treated^52,63,64^. The replacement of fossil fuels with RDF encourages the establishment of a circular economy, decreases landfill reliance, and favors climate promises^65^. Nevertheless, in part areas, thermal replacement rate of RDF is small because it is not easy to satisfy a certain requirement of heat value^64^.

The financial evaluations always prove that production and use of RDF in the cement sectors can produce net economic benefits to municipalities, consumers, and the society in general^15,63^. The cost saving comes due to less landfill prices, less fuel cost, and, in some situations, RDF revenue^15,66^. As an illustration, in Macedonia, the price of investments and operations costs per ton were stated as 21.67 $ ton^− 1^ and 5.04 $ ton^− 1^, respectively, and the feasibility was limited by the transport cost to the cement plant^66^. In Indonesia, it is possible to save up to 421 million $ annually in case RDF were to substitute a part of coal in producing cement^67^.

#### Technical feasibility and operational considerations

However, economic viability is determined by such elements as capital spending, coal costs, landfill costs, and the dependability of the RDF supply and quality^67,68^. Data shown by both the pilot- and industrial-scale trials indicate that even large percentages of RDF fractions, or more specifically over 90% using an energy basis, should be able to be used effectively, especially when used in conjunction with other alternative energy sources like hydrogen without negatively affecting the combustion efficiency^67^.

Moisture content and calorific value of RDF are of paramount importance to the efficiency of the operations with moisture content being advised to be less than 15% in order to realize effective carbon offset^69^. Bio-drying and palletization are pre-treatment processes that result in increased energy content in RDF and cement kiln viability^68,70^. This study reveals findings consistent with previous studies in that net economic benefits fall within a 13.2–58.3 $ ton^− 1^ of cement at Titan cement facility because RDF is one of the most financially beneficial strategies.

The net savings of 12.033 $ ton^− 1^ of cement using RDF of the Nahdet Misr MSW Treatment Facility is economically feasible even at medium levels of substitutions. In addition, local RDF mitigates the use of foreign coal, where cost savings will be between 11 and 19.8 $ ton^− 1^ cement in accordance to the calorific value of coal and market dynamics. In the case of Titan cement plant, a reduction in imported coal will increase the economic benefits further and the estimated coal savings will be between 16.5 and 27.5 $ ton^− 1^ of cement, reduction of CO_2_ will be between 8.8 and 16.5 $ ton^− 1^ of cement and RDF gate-fee will be between 16.5 and 33 $ ton^− 1^ of RDF.

### Summary and implications

To sum up, a higher percentage of RDF content in cement production can be advantageous to a greater degree in the three aspects of environmental, economic, and resource-saving, at the same time promoting the concept of the circle economy. LCA research has verified that GHG emissions, acidification, eutrophication and reduction in landfills are reduced, with indicators of human health improved. The benefits however, can be realized only when the issues of operation and technical control are handled with care, such as keeping emissions down, system efficiency and fuel quality. However, it is possible to make the cement industry effectively advance to environmentally friendly production by considering these factors with the help of a strong system analysis and relevant technological solutions^50,52,64^.

## Conclusions

Conclusions Life cycle environmental assessment gives strong arguments that the use of fossil fuels can be partially substituted with refuse-derived fuel (RDF) in cement production; this is a feasible approach to carbon emission reduction and support of the established goals of the circular economy. Although environmental and economic advantages of using RDF are significant, attaining the most appropriate results should be paid close attention to the quality of fuels, the optimization of the process, and the peculiarities of the local environment.

This experiment involved LCA to estimate the environmental performance of RDF obtained out of MSW in 5 cement manufacturing conditions namely Scenario I (0% RDF), Scenario II (20% RDF), Scenario III (50% RDF), Scenario IV (80% RDF), Scenario V (100% RDF). The findings show that Scenario V has the best environmental sustainability as compared to Scenario I. Key environmental impact indicators for Scenario V include: marine aquatic ecotoxicity, 125052.98 kg 1,4-DPB eq; human toxicity, 72.05 kg 1,4-DB eq; freshwater aquatic ecotoxicity, 35.3 kg 1,4-DB eq; photochemical oxidation, 0.08033 kg C₂H₄ eq; abiotic depletion of fossil fuels, 1109.73 MJ; acidification, 1.67 kg SO₂ eq; total abiotic depletion, 3.6E-04 MJ; global warming potential (GWP₁₀₀a), 858.18 kg CO₂ eq; and eutrophication, 3.1E-01 kg PO₄ eq.

The gradual replacement of RDF would obviously have both environmental and financial benefits, yet to achieve its success, the technical, operational, and pollution management issues will need to be resolved with the consideration of the proper regulatory framework and infrastructure, as well as through the optimization of fuel quality and combustion conditions.

## Data Availability

All data included in the research will be made available upon request, while the data from previous studies and research was obtained through the Cairo University platform, which provides research information on a regular basis. If someone wants to request the data from this study, kindly contact Prof. Dr. Mohamed E. Abuarab (mohamed.aboarab@agr.cuedu.eg).
